# In Vivo Neurodynamics Mapping via High‐Speed Two‐Photon Fluorescence Lifetime Volumetric Projection Microscopy

**DOI:** 10.1002/advs.202410605

**Published:** 2024-12-23

**Authors:** Yanping Li, Xiangcong Xu, Chao Zhang, Xuefeng Sun, Sisi Zhou, Xuan Li, Jiaqing Guo, Rui Hu, Junle Qu, Liwei Liu

**Affiliations:** ^1^ State Key Laboratory of Radio Frequency Heterogeneous Integration & Key Laboratory of Optoelectronic Devices and Systems College of Physics and Optoelectronic Engineering Shenzhen University Shenzhen 518060 China

**Keywords:** frequency‐domain fluorescence lifetime microscopy, high‐speed volumetric projection imaging, in vivo neurodynamics

## Abstract

Monitoring the morphological and biochemical information of neurons and glial cells at high temporal resolution in three‐dimensional (3D) volumes of in vivo is pivotal for understanding their structure and function, and quantifying the brain microenvironment. Conventional two‐photon fluorescence lifetime volumetric imaging speed faces the acquisition speed challenges of slow serial focal tomographic scanning, complex post‐processing procedures for lifetime images, and inherent trade‐offs among contrast, signal‐to‐noise ratio, and speed. This study presents a two‐photon fluorescence lifetime volumetric projection microscopy using an axially elongated Bessel focus and instant frequency‐domain fluorescence lifetime technique, and integrating with a convolutional network to enhance the imaging speed for in vivo neurodynamics mapping. The proposed method is validated by monitoring intracellular Ca^2+^ concentration throughout whole volume, tracking microglia movement and microenvironmental changes following thermal injury in the zebrafish brain, analyzing structural and functional variations of gap junctions in astrocyte networks, and measuring the Ca^2+^ concentration in neurons in mouse brains. This innovative methodology enables quantitative in vivo visualization of neurodynamics and the cellular processes and interactions in the brain.

## Graphical abstract

Two‐photon fluorescence lifetime microscopy has made tremendous inroads toward monitoring the morphological and biochemical information of central nervous system. In this paper, we demonstrate a high‐speed volumetric two‐photon fluorescence lifetime microscopy that combines the TP‐FD‐FLIM technique with Bessel‐based volumetric excitation and machine learning‐based enhancement methods to overcome traditional limitations in speed, contrast, and SNR imaging. This technology provides a powerful tool for observing brain cell dynamics and monitoring the brain microenvironment in live organisms.

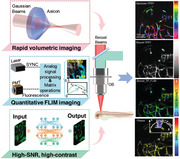

## 1 Introduction

Studying the interactions and communication between neurons and glial cells in the central nervous system and quantitatively monitoring the brain microenvironment is crucial for understanding the intricate relationship between cellular activities and local metabolism. This insight is fundamental to advancing knowledge in neuroscience and developing effective treatments for brain diseases.^[^ ^1^ ^]^ Two‐photon (TP) microscopy combined with fluorescence probes has become a popular alternative to electrophysiological techniques for studying neurodynamics.^[^ ^2^, ^3^, ^4^ ^]^ However, the fluorescence intensity is susceptible to several factors (photobleaching, fluorophore concentration, background signal, and excitation light source power drift), complicating accurate quantitative analysis. Fluorescence lifetime, a significant parameter, contains a wealth of information about the molecular microenvironment (e.g., pH, refractive index, ion concentration, and dissolved gas concentration) and mitigates the adverse effects of excitation light intensity, fluorophore concentration, and environmental factors.^[^ ^5^, ^6^, ^7^, ^8^ ^]^ Thus, fluorescence lifetime imaging microscopy (FLIM) combined with multiphoton‐excited fluorescence (MPEF)^[^ ^9^ ^]^ offers a powerful approach for high‐resolution deep‐tissue quantitative imaging with reduced photodamage.^[^ ^10^ ^]^

While FLIM has significant advantages and potential for neurodynamic studies,^[^ ^11^, ^12^, ^13^, ^14^ ^]^ its application in vivo is hindered by slow acquisition rates.^[^ ^15^, ^16^, ^17^ ^]^ The time‐correlated single‐photon counting (TCSPC) method, widely used for fluorescence lifetime detection, constructs a histogram to estimate the fluorescence decay profile by recording the arrival times of each photon repetitively (at least 10 µs/pixel), and requires complicated post‐processing procedures.^[^ ^18^ ^]^ Although analog mean‐delay (AMD)^[^ ^19^ ^]^ and the center‐of‐mass method (CMM)^[^ ^20^ ^]^ minimize the pixel dwell time, their extensive data acquisition and signal processing still hinder the continuous monitoring of dynamic processes. The frequency‐domain fluorescence lifetime microscopy (FD‐FLIM), based on a modulation–demodulation technique, can achieve lifetime image acquisition and processing as fast as 40 µs/pixel.^[^ ^21^ ^]^ Although siFLIM, equipped with a dedicated camera for phase detection,^[^ ^22^ ^]^ enables video‐rate (approximately ten frames per second) lifetime imaging of living cells, its dependence on widefield microscopy prevents volumetric lifetime imaging.

Yet, capturing the comprehensive multiparameter properties (structure, function, and microenvironment) of cells and tissues in vivo within 3D volumes is essential for understanding their physiological processes, necessitating high‐throughput and high‐speed volumetric fluorescence lifetime imaging for in vivo neurodynamics research.^[^ ^23^, ^24^, ^25^, ^26^, ^27^, ^28^ ^]^ Instant FD‐FLIM, with high‐speed image acquisition and processing capabilities, allows real‐time streaming of 2PEF intensity, lifetime, and phasor results for volumetric lifetime imaging in vivo.^[^ ^17^ ^]^ Nevertheless, when using Gaussian beams, instant FD‐FLIM is done over thin 2D slices, relying on axial scanning at multiple focal planes to form a z‐stack. This method increases acquisition time, rendering capturing dynamic events challenging. Other fundamental issues, including low contrast, low signal‐to‐noise (SNR) ratio, and higher lifetime measurement error,^[^ ^29^ ^]^ are common in deep living tissue imaging. Conventional denoising techniques, such as averaging (mean filtering) or median weight filtering, improve the SNR but result in image blurring and reduced frame rates with substantial computational overhead.^[^ ^30^ ^]^ Deep learning algorithms^[^ ^29^, ^31^ ^]^ offer a powerful solution for enhancing image contrast and SNR without compromising high‐speed acquisition. These algorithms effectively reduce noise and improve clarity in challenging conditions (such as low power light excitation or short exposure times), preserving image quality while minimizing photodamage and maximizing acquisition speed.

To address these challenges, we developed a high‐speed two‐photon fluorescence lifetime volumetric projection microscope, utilizing a Bessel beam for excitation and enhanced through a deep learning algorithm, specifically to meet the needs of in vivo neurodynamics fluorescence lifetime imaging. This method improves imaging speed through two perspectives of signal excitation and information processing by integrating rapid volumetric excitation technology based on extended depth of field (DOF) with efficient fluorescence lifetime extraction method. Additionally, the deep learning framework addresses issues of low image contrast and SNR for high‐speed and high‐throughput fluorescence lifetime imaging. This innovated method provides a powerful tool to study the interaction and communication within the central nervous system, including neurons and glial cells, and to quantitatively monitor the brain microenvironment.

In this work, we proved the capability of the high‐speed two‐photon fluorescence lifetime volumetric projection microscopy to monitor the changes in intracellular Ca^2+^ concentration under drug stimulation. We further demonstrated the movement trajectory of microglia during the repair process after thermal injury in the zebrafish brain, dynamically and quantitatively characterizing the tissue microenvironment during this process. Additionally, we investigated the microenvironmental variation of gap junctions in astrocyte networks and the Ca^2+^ concentration of neurons in mouse brains, validating the potential of this method for quantitatively visualizing in vivo neurodynamics.

## 2 Results

### 2.1 Experimental Design and TP‐FD‐FLIM Volumetric Projection Microscopy

**Figure 1:**
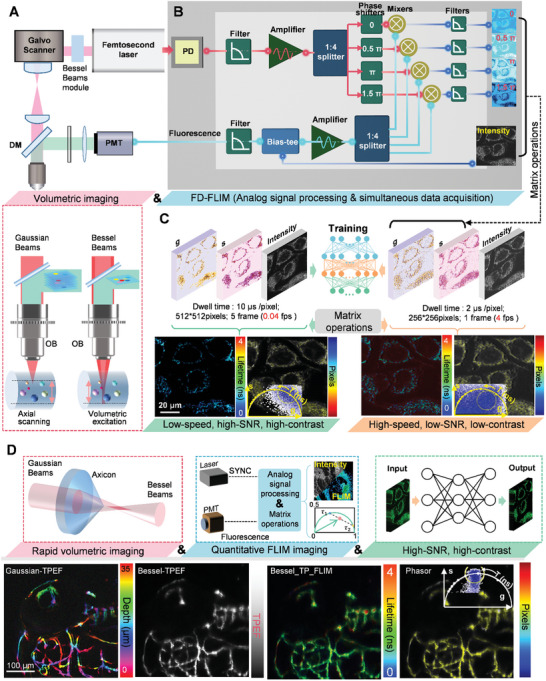
Experimental Design and Workflow. A) Brief diagram of the TP‐FD‐FLIM volumetric projection microscopy, including a custom‐built two‐photon laser scanning microscope, a rapid volumetric excitation module, and an FD‐FLIM demodulation module. B) Overview of the analog signal processing, simultaneous signal acquisition, and matrix operation processing of intensity, lifetime, and phasor data during FD‐FLIM demodulation. C) Raw intensity, *g*, and *s* images with low resolution, contrast, and SNR are used as input data to train the deep learning network. The corresponding high‐quality images are used as target data. After training, a denoising model can be established and memorized in the network parameters. D) Gaussian two‐photon intensity projection image (color‐coded by depth) of zebrafish brain vasculature expressing enhanced green fluorescent protein (eGFP), and corresponding Bessel volumetric projection imaging results enhanced by the deep learning network (including two‐photon intensity, FLIM images, phasor plot, and phasor‐labeled image). The image frame rate is 2 fps. PD: photodiode; PMT: photomultiplier tube; DM: dichroic mirror.

This study aimed to quantitatively analyze rapid neurodynamic processes for in vivo brain research using high‐speed two‐photon frequency‐domain fluorescence lifetime volumetric projection microscopy (TP‐FD‐FLIM). To address the limitations of slow image acquisition in TCSPC‐based TP‐FLIM platforms and the complexities involved in monitoring dynamic processes within living microenvironments, we constructed an FD‐FLIM system that employs homodyne detection theory and hardware analog circuit signal processing methods. This system instantaneously captures the real‐time two‐photon intensity, fluorescence lifetime, and phasor plot information through synchronous signal acquisition and matrix operations (**Figure** **1**B, Experimental Section, and Note S1, Supporting Information). Additionally, we introduced Bessel beams with an extended DOF, superseding Gaussian beams for volumetric excitation (Figure 1A), thereby resolving the issue of slow axial tomographic scanning in Gaussian‐beam‐based volumetric imaging. The excited axial length of the Bessel beam (35 µm) was approximately fourteen times greater than that of a Gaussian beam (2.5 µm) focused by a 20×, 0.75NA objective, and both beams achieved nearly the identical lateral resolution (≈550 nm). A detailed performance evaluation was provided in Note S4 (Supporting Information). The superiority of Bessel‐beam‐based high‐throughput volumetric projection imaging was further discussed in Note S6 (Supporting Information), and the comparison results of fluorescence intensity and lifetime excited by Gaussian and Bessel beam in in vivo imaging were shown in Figures S7 and S8 (Supporting Information). The integration of FD‐FLIM with Bessel‐beam excitation enables rapid fluorescence lifetime volumetric projection imaging in vivo. Furthermore, we optimally resolved the trade‐off between the capture velocity and quality of the fluorescence intensity and lifetime maps using a deep‐learning framework, achieving a state‐of‐the‐art improvement that increased the acquisition speed by a factor of 100 (Figure 1C). Our study combined Bessel volumetric excitation technology, an instant FD‐FLIM setup, and deep‐learning networks for fluorescence intensity and lifetime volumetric projection imaging of in vivo features, enhancing contrast, SNR, and running time advantages (Figure 1D). The simultaneously obtained phasor plot can be used for tissue structure segmentation and component analysis.

For FD‐FLIM volumetric projection platform, we utilized a flexible axicon‐based Bessel module, which allowed for the generation of Bessel beams with extended axial ranges during two‐photon excitation imaging. This approach enabled us to capture 2D projection images of 3D volumetric specimens. Figure S1 (Supporting Information) provides a simplified diagram of the TP‐FD‐FLIM volumetric projection optical system, based on a custom‐built two‐photon laser scanning microscope. Analog signal processing plays a crucial role in the TP‐FD‐FLIM volumetric projection system. The fluorescence signal and reference signal from the femtosecond laser are used to simultaneously generate the intensity, lifetime, and phasor data. In particular, the femtosecond excitation pulse is detected by custom‐built photodetectors with a 100 MHz bandwidth, serving as the reference signal. The two‐photon excitation fluorescence signal is detected by a photomultiplier tube (PMT) equipped with a transimpedance amplifier. The output voltage signal is filtered by a low‐pass filter (LPF) and separated into DC and radio frequency (RF) components using a bias tee. The RF signal and the reference signal at 80 MHz are amplified using low‐noise amplifiers (LNA) and split in four paths via four‐way power splitters. Subsequently, each split laser reference signals, with different phases introduced by four phase shifters, is then mixed with the four‐way RF signal. The mixer outputs, filtered by the LPF and the bias tee's DC part, are digitized simultaneously by a data acquisition (DAQ) card. The components of the phasors, fluorescence lifetime images, and phasor plots are generated in real‐time through basic matrix operations. DAQ cards are also employed to control the galvo scanner during the imaging process. A comprehensive description of the analog signal processing module, including device parameters and the simultaneous acquisition and processing of intensity, lifetime, and phasor data, is provided in Materials and Methods, Figure S2 and Note S2 (Supporting Information).

Fluorescence microscopy imaging inevitably introduces a combination of Poisson–Gaussian noise,^[^ ^31^ ^]^ leading to raw intensity and FLIM data with typically low SNR, and the phasor plot with inaccurate clustering, making fluorophores identification challenging. Although increasing the pixel dwell time and averaging multiple images can effectively improve image contrast and SNR, these methods reduce the frame rate, making them unsuitable for live dynamic monitoring. We implemented a convolutional network inspired by a U‐shaped network (U‐Net) (Experimental Section) to achieve high‐speed imaging, with improved contrast and SNR. As lifetime maps and phasor plots used for biological mechanism analysis can be derived from intensity images and phasor components (*g* and *s*), we employed a deep learning model to separately enhance the quality of these images. The enhanced output images were then processed by the FLIM analysis program to generate additional FLIM data and phasor plots. To train the model, we used HeLa cells labeled with mitochondrial probes to obtain the intensity, *g*, and *s* images. The input images were captured with a short dwell time (2 µs/pixel) at 4 fps, resulting in a frame time of 250 ms for 256 × 256 pixels. The target high‐quality images, approximating the ground truth (GT), were acquired with a long dwell time (10 µs/pixel) at 0.04 fps, over 25 s with 5 frames for 512 × 512 pixels. After training and optimization, the deep learning model generated intensity, *g*, and *s* images with high resolution, contrast, and SNR, allowing for the calculation and production of high‐quality FLIM images and phasor plots.

### 2.2 Analysis of Image Quality Improvement

**Figure 2:**
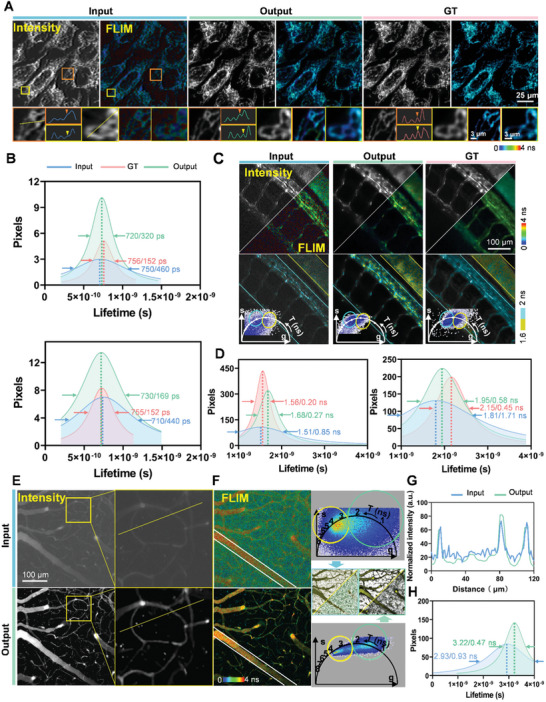
Deep learning‐powered TP‐FD‐FLIM volumetric projection microscopy. A) Fluorescence intensity and lifetime images of mitochondria labeled with MitoTracker Red in HeLa cells, including input, network output, and GT results. Enlarged views of rod‐like and network‐like mitochondria are highlighted within the orange and yellow blocks. The profile curves display the intensity variation at the position of the yellow dashed line. B) Histogram of fluorescence lifetime distribution for the orange (above) and yellow (below) block areas in (A). C) Bessel‐based volumetric projection images of zebrafish brain vasculature expressing eGFP, including two‐photon intensity, FLIM images, phasor plot, and phasor‐labeled images. D) Histogram of fluorescence lifetime distribution for the yellow (left) and blue (right) regions in (C). Bessel‐based volumetric projection imaging results of mouse brain blood vessels labeled with FITC, including E) two‐photon intensity, and F) FLIM images, phasor plot, and phasor‐labeled images. G) Intensity profile along the yellow dashed line in the enlarged areas of (E). H) Histogram of the fluorescence lifetime distribution within the white dashed regions in (F).

We designed a U‐Net‐based framework for FD‐FLIM, enhancing image reconstruction by integrating convolutional block attention modules (CBAM) into each layer of the U‐Net. The encoder‐decoder architecture captures detailed fluorescence lifetime information within the encoder, and while the decoder, aided by skip connections, ensures precise image localization. To achieve robust feature learning on the variable *g* dataset, we employed contextual bilateral (CoBi) loss, while perceptual loss was used to improve visual accuracy for the *s* and intensity datasets. Optimization was carried out using the Adam optimizer with a cosine learning rate scheduler over 500 epochs, minimizing the mean absolute error (MAE) to ensure high fidelity. Image augmentation techniques, such as flipping and rotation, further bolstered model robustness. We utilized metrics such as peak signal‐to‐noise ratio (PSNR), structural similarity index measure (SSIM), root mean squared error (RMSE), and MAE to quantitatively evaluate the performance of our deep learning network (Figure S6 and Note S5, Supporting Information). Additionally, the mean squared error (MSE) and phasor representation of the processed fluorescence lifetime distribution provided a relative quantification assessment of denoising effectiveness. A comparison between the registered input image with 256 × 256 pixels, the network output image with 512 × 512 pixels, and the GT image of mitochondria labeled with MitoTracker Red in HeLa cells was shown in **Figure** **2**A. Overall, qualitative mitochondrial morphological features, including rod‐like structures (orange block in Figure 2A) and network‐like (yellow block in Figure 2A) structures, were well‐preserved in the network output but appeared blurry in the input image. The intensity curve reveals that the input image was impaired by significant background noise and low contrast, resulting in the loss of many details (denoted by the arrowhead). Conversely, the network output exhibits high image contrast, enabling the clear discrimination of detailed mitochondrial features, which are nearly identical to those in the GT image. Additionally, the histogram of the fluorescence lifetime distribution quantitatively indicates that the noise in the *g*, *s*, and intensity images was highly suppressed by the proposed deep learning network. The peak values and MSE of the lifetime data distributions (input of 750/460 ps, output of 720/320 ps, and GT of 756/152 ps for the orange block; and input of 710/440 ps, output of 730/169 ps, and ground truth of 755/152 ps for the yellow block) confirm the fidelity of the network output. These results demonstrate that the deep learning network effectively enhances the SNR of FLIM images (Figure 2B).

We also applied the deep learning network to in vivo imaging of zebrafish vasculature labeled with eGFP (*flila: eGFP*) and mouse brain blood vessels marked with fluorescein isothiocyanate (FITC) to further verify the performance of TP‐FD‐FLIM volumetric projection imaging powered by the network. Figure 2C presents Bessel‐based volumetric projection images of the zebrafish vasculature, including intensity, lifetime, and phasor‐labeled information, along with the corresponding transformation results. The high background noise in the intensity maps, stray lifetime distribution in the lifetime images, and inaccurate clustering in the phasor plots of the input images were improved in the output results. In particular, the cluster segmentation in the output phasor plot, was significantly enhanced, exhibiting high contrast and SNR. This allowed for precise mapping of the vasculature labeled with eGFP and autofluorescence in vivo. The MSE of the input lifetime data was notably reduced, whereas the peak value results retained within acceptable error range, as demonstrated by the histogram in Figure 2D. We further improved the volumetric projection imaging of in vivo mouse brain blood vessels (depth: 200 µm, DOF: 35 µm). The fluctuations in the intensity profile of the input image (Figure 2E) were considerably reduced in the output results, while morphological information was preserved and denoised, as shown by the cross‐sections in Figure 2G. Fine microstructures with low contrast that were previously obscured by background noise in the input lifetime image were greatly enhanced in the output lifetime image (Figure 2F). The fluorescence lifetime distribution histogram (Figure 2H) quantitatively demonstrates the enhancement of SNR in the processed fluorescence lifetime image. Additionally, a small fraction of FITC infiltrates from cerebral blood vessels into the surrounding brain tissue caused detectable changes in fluorescence lifetime due to microenvironment difference between the vasculature and tissue, which were precisely segmented by improved cluster boundary of the output phasor plot.

### 2.3 Dynamic Monitoring of Intracellular Ca^2+^ Concentrations

**Figure 3:**
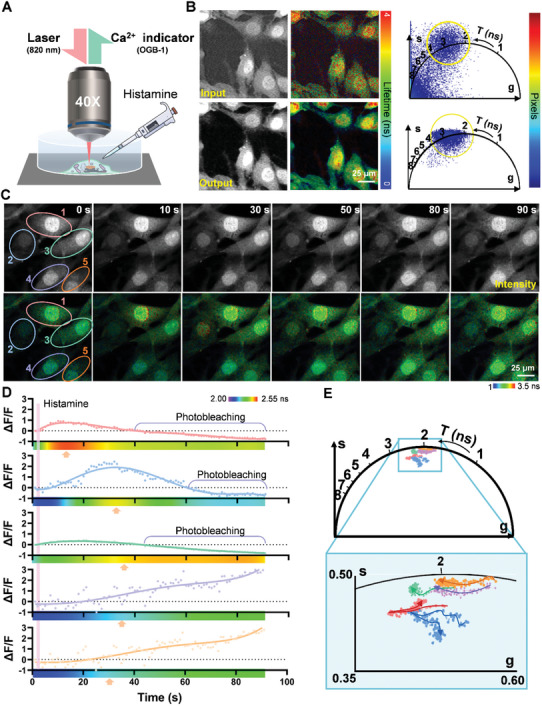
Dynamic monitoring of Ca^2+^ concentration in cultured cardiomyocytes. A) Ca^2+^ levels in cells within a petri dish were measured using a femtosecond pulse laser with a wavelength of 820 nm (for OGB‐1) and a 40× objective, with histamine stimulation applied at *t* = 2 s. The sampling rate was set to 1 Hz. B) Comparison of input and network output, including fluorescence intensity, lifetime images, and phasor plots. C) Time‐series results displaying fluorescence intensity and lifetime of Ca^2+^ levels over 90 s. D) Time‐dependent curves of fluorescence intensity and kymographs of TP‐FD‐FLIM volumetric projection imaging values for the five cells indicated by the circle in (C). Arrowheads highlight elevated Ca^2+^ levels as characterized by changes in lifetime values. E) Temporal trajectory of fluorescence lifetime distribution presented in the phasor plot across the five cells within the circle in (C).

Dynamic fluctuations in intracellular Ca^2+^ concentrations drive essential signaling cascades in neurocytes,^[^ ^32^, ^33^ ^]^ including axonal neurotransmitter release, dendritic regulation of synaptic plasticity, and communication within electrically passive astrocytes. In situ monitoring of Ca^2+^ level is typically conducted using fluorescence intensity‐based measurements with fluorescent Ca^2+^ indicators. While this method is effective for capturing significant and rapid Ca^2+^ level fluctuations, its accuracy at low Ca^2+^ levels is constrained by errors related to low SNR, fluctuations in dye concentration, focus drift, and photobleaching.^[^ ^34^, ^35^ ^]^ In contrast, FLIM techniques remain unaffected by these factors that impede traditional fluorescence imaging. The fluorescence lifetime of one of Oregon Green BAPTA‐1(OGB‐1), a common Ca^2+^ indicator, is sensitive to Ca^2+^ concentrations and remains unaffected by physiological changes in pH, concentrations of Mg^2+^ and Zn^2+^, temperature, and microviscosity.^[^ ^12^, ^13^ ^]^ Consequently, the high‐speed two‐photon fluorescence lifetime volumetric projection microscopy in this study allows for monitoring Ca^2+^ concentration fluctuations within the entire live cell (a volumetric range with DOF of 15 µm) rather than just local Ca^2+^ concentration during histamine stimulation, as assessed through lifetime measurement, facilitating the study of long‐term global cellular responses (**Figure** **3**A).

We initially validated the fidelity and superior performance of our deep learning model for imaging intracellular Ca^2+^ concentration (Figure 3B). The output image with high contrast and SNR enables accurate quantification of Ca^2+^ level and precise phasor clustering to identify fluorescent clusters (see the full input, output, and GT images in Figure S9, Supporting Information). To demonstrate the efficacy of rapid TP‐FD‐FLIM volumetric projection microscopy, we conducted 90‐s imaging sequences of Ca^2+^ levels in cardiomyocytes at a frame rate of 1 Hz (Figure 3C; Movie S1, Supporting Information). Following histamine addition, intracellular Ca^2+^ levels rose at various time points, triggered by the interaction between histamine and its specific receptors, which regulate Ca^2^⁺ concentrations via distinct signaling pathways.^[^ ^36^ ^]^ We analyzed the fluorescence intensity and lifetime of five cells across the entire field of view (FOV), as depicted in Figure 3D. While fluorescence intensity images effectively illustrate variations in Ca^2+^ concentration, photobleaching significantly hampers intensity measurement during time‐series imaging, rendering accurate monitoring of Ca^2^⁺ concentration challenging. Additionally, we present the temporal trajectory of the phasor distribution across different cells (Figure 3E) to analyze the attribution of phasor components in intracellular Ca^2+^ variations.

### 2.4 Dynamic Functional Imaging of Microglia in Zebrafish

**Figure 4:**
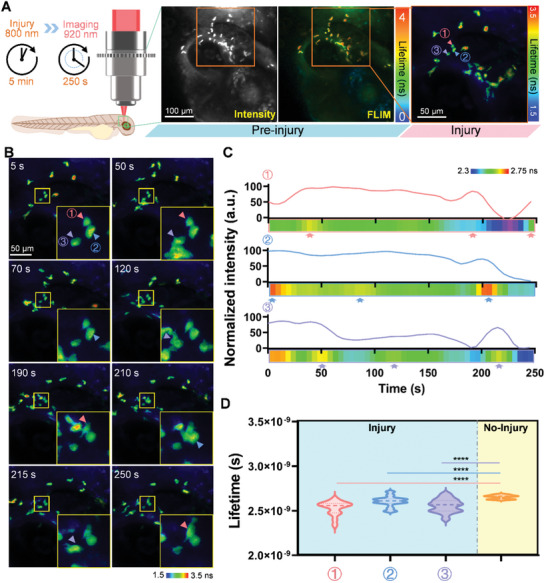
Dynamic functional imaging of microglia in zebrafish. A) Larval *Tg(pu1: eGFP)* zebrafish, at 4 days post‐fertilization, were imaged using TP‐FD‐FLIM volumetric projection microscopy following an injury induced by an 800 nm femtosecond laser for 5 min. B) Z‐projection images of volumetric TPEF lifetime was obtained from a 250 s FLIM movie of microglia in the zebrafish brain post‐laser injury. Arrowheads indicate region of interest (ROI) where microglia approached one another. C) Time‐varying fluorescence intensity curve and fluorescence lifetime kymograph are shown for the ROI cells in (A) and (B). D) Statistical analysis of differences in the fluorescence lifetime of microglia cells before and after injury (*n* = 50). An unpaired Mann‐Whitney test was applied, and the mean ± SD is shown in (D), ****: *p* < 0.001.

We further validated the system's capability to explore the central nervous system in vivo by characterizing intricate biological processes and microenvironmental information within living organisms. Microglia, as active sensors and specialized phagocytes, are exceptionally versatile immune cells in the central nervous system. They continuously monitor their microenvironment through dynamic processes^[^ ^37^, ^38^ ^]^ and are highly sensitive to a range of central nervous system disturbances, including pathogens, danger signals, and neurotransmitters.^[^ ^39^ ^]^ Moreover, microglia play a crucial role in shaping neural network patterns and regulating other glial cells and angiogenesis. Consequently, dynamic functional imaging of microglia holds significant promise for elucidating brain neural regulation and understanding the mechanisms underlying neurological diseases.^[^ ^40^, ^41^ ^]^ We assessed the in vivo performance of TP‐FD‐FLIM volumetric projection microscopy by measuring fluorescence intensity, lifetime, and phasor plots of microglia expressing eGFP within the 3D volume (400 µm × 400 µm × 35 µm) of living zebrafish (**Figure** **4**A). This approach provides global coverage and continuity of tissue structures, facilitating systematic research on physiological changes.

To test the Bessel‐based TP‐FD‐FLIM system's capability for dynamic functional imaging in vivo, we first imaged *Tg(pu1: eGFP)* zebrafish, which express eGFP in microglia, at 3 days post‐fertilization. We continuously monitored the microglia for 250 s, capturing frames at a rate of 0.2 fps. In the absence of external stimulation or tissue damage, the microglia in the zebrafish brain displayed small‐scale movements (Movie S2, Supporting Information), with cell bodies remaining largely stationary. Minor scanning motions were observed at branching sites to monitor the surrounding environment, and the average fluorescence lifetime of the microglia remained stable during homeostasis. We then examined lifetime changes in microglia in response to thermal brain injury, induced by a high power (40 mW) 800 nm femtosecond laser applied for 5 mins. Instant FLIM movies, composed of time‐dependent volumetric projection microglia maps stacks, were captured at 0.2 fps for 250 s (Figure 4B; Movie S3, Supporting Information). Consistent with injury responses,^[^ ^38^, ^42^ ^]^ microglia were rapidly activated and migrated to regulate inflammation and tissue repair. The fluorescence intensity movie revealed the movement trajectory of the microglia, yet, time‐dependent intensity variations (Figure 4C) were affected by photobleaching, axial fluorescence signal integration, and focal drift, complicating further analysis of damage repair mechanisms. Fluorescence lifetime results provided additional insights into the microenvironment. Analysis of the fluorescence lifetime kymograph (Figure 4C) for the three microglia marked in Figure 4A,B indicated a downward trend in fluorescence lifetimes for eGFP expression in microglia following increased temperature.^[^ ^17^ ^]^ Additionally, the lifetime value increased instantly (indicated by arrows) when other microglia approached each other. This phenomenon is likely due to the release of cytokines (secreted proteins) during microglial damage repair,^[^ ^41^ ^]^ which affects the fluorescence lifetime of eGFP expressed in microglia.^[^ ^43^ ^]^ Statistical analysis (Figure 4D) of the fluorescence lifetime data revealed significant differences (*p* < 0.001) between pre‐ and post‐injury states. These observations demonstrate the capacity of TP‐FD‐FLIM volumetric projection microscopy for dynamic function monitoring in vivo following physiological challenges.

### 2.5 Astrocytes Functional Imaging and Spontaneous Calcium Dynamics in Mice Brain

**Figure 5:**
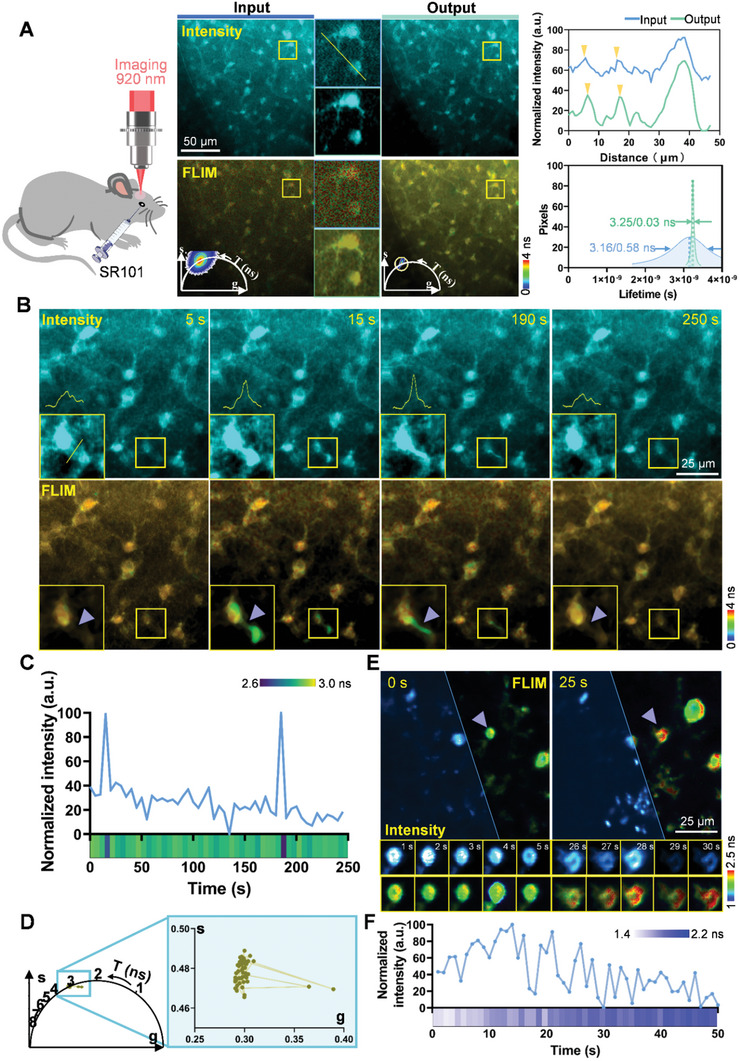
Astrocytes functional imaging and spontaneous calcium dynamics in mice brain. A) Fluorescence intensity and lifetime images, along with the phasor plot, of astrocytes labeled with SR101 in the mouse brain. The intensity profile along the yellow line in the enlarged image and the fluorescence lifetime histogram of the enlarged lifetime image demonstrate the effectiveness of the network in enhancing contrast and SNR. B) Z‐projection images of volumetric TPEF intensity and lifetime from a 250 s FLIM movie of astrocytes in the mouse brain. The intensity profile curve of gap junctions, indicated by the yellow line, is shown in the bottom left corner of the ROI. C) Time‐dependent fluorescence intensity curve and lifetime kymograph of gap junctions over 250 s, revealing the opening and closing of gap junctions. D) Temporal trajectory of the average fluorescence lifetime distribution in the phasor plot for gap junctions in astrocytes. E) Intensity and lifetime images of Ca^2+^ in neurons within the mouse cortex, with the magnified neuron (denoted by purple arrowheads) shown at various time points. F) Time‐dependent fluorescence intensity curve and lifetime kymograph of the magnified neuron over 50 s, illustrating spontaneous neural activity.

We explored the capability of high‐speed two‐photon fluorescence lifetime volumetric projection microscopy to image astrocyte functionality and spontaneous calcium dynamics in mice, validating its effectiveness for neurodynamics imaging (**Figure** **5**). Rapid fluorescence intensity and lifetime volumetric projection imaging yielded lower contrast and SNR due to increased tissue scattering in vivo. Consequently, we first evaluated the suitability of the network used in this study for astrocyte imaging. Compared to the original input, the output intensity images restored the finer details of astrocytes successfully, the fluorescence lifetime results exhibited a more compact phasor distribution with minimal stray lifetime (3.25/0.03 ns), as shown in Figure 5A.

Astrocytes play essential roles in synaptic function, brain activity, and animal behavior within the central nervous system.^[^ ^44^ ^]^ Due to their high sensitivity to photoactivation, prolonged exposure to high laser power significantly impact their physiology.^[^ ^45^, ^46^, ^47^ ^]^ To mitigate this effects, we monitored the fluorescence intensity and lifetime of astrocytes labeled with Sulforhodamine 101 (SR101) over 250 s, capturing frames at a rate of 0.2 fps. During this period, continuous laser excitation led to physiological microenvironment alterations, evidenced by fluctuations in both fluorescence intensity and lifetime (Figure 5B; Movie S4, Supporting Information). Specifically, the fluorescence intensity (Figure 5C) of gap junctions, composed primarily of connexins (Cxs), exhibited transient spikes at 15 and 190 s within the astrocyte network, likely due to the changes in the physiological environment causing the opening and closing of gap junctions.^[^ ^48^, ^49^ ^]^ At these points, we observed an immediate reduction in the fluorescence lifetime of SR101 expressed in gap junctions (Figure 5C), indicating sensitivity to physiological factors such as ion concentration and pH, unlike the intensity‐based reading. The phasor plot (Figure 5D) revealed that the alteration in average fluorescence lifetime was primarily due to an increase in the *g*‐phase component, providing insights into excited‐state reactions (e.g., dipolar relaxation, FRET acceptor emission, and excimer formation).^[^ ^50^ ^]^

Next, we validated the performance of TP‐FD‐FLIM volumetric projection microscopy under challenging conditions by capturing Ca^2+^ signals (both intensity and lifetime) from neurons in the mouse cortex at a frequency of 1 Hz to monitor rapid Ca^2+^ dynamics in vivo (Figure 5E; Movie S5, Supporting Information). Individual neurons exhibiting spontaneous activity were isolated and magnified in Figure 5E. These neurons displayed a drift pattern attributable to respiratory tremors, which compromised the accuracy of fluorescence intensity signals (Figure 5F) in tracking changes in Ca^2+^ concentration. In contrast, fluorescence lifetime measurements, less susceptible to environmental fluctuations, provided a more reliable indication of Ca^2+^ concentrations changes during in vivo spontaneous neural activity.

## 3 Discussion

In summary, by integrating the TP‐FD‐FLIM technique with Bessel‐based volumetric excitation and machine learning techniques, we have advanced high‐speed two‐photon fluorescence lifetime volumetric projection microscopy, effectively overcoming traditional limitations in speed, contrast, and SNR. Our approach enables the rapid acquisition of high‐resolution, high‐contrast fluorescence intensity and lifetime images, as well as lifetime phasor component information in 3D volumetric organisms. The novel aspects of volumetric TP‐FD‐FLIM include: 1) real‐time fluorescence intensity and lifetime acquisition using FD‐FLIM based on four‐phase analog signal processing, which replaces the long‐term photon accumulation and complex post‐processing procedures of TCSPC‐based TP‐FLIM; 2) rapid volumetric projection imaging using Bessel beams for excitation, which solves the slow axial tomographic scanning associated with Gaussian beams; and 3) the application of a deep learning algorithm to balance the inherent trade‐off between imaging speed and contrast, SNR.

On the application side, our methods facilitate the quantitative investigation of intracellular Ca^2+^ concentration changes under drug stimulation through high‐speed image acquisition. Additionally, polar plot analysis can serve as an auxiliary tool to interpret the mechanisms regulating Ca^2+^ concentration. The advantages of the TP‐FD‐FLIM volumetric projection platform have been extensively demonstrated through in vivo neurodynamic imaging of zebrafish and mouse brains. We reveal the movement trajectory and microenvironmental changes of microglia in the zebrafish brain before and after thermal damage, illustrating how dynamic functional imaging can assist biologists in understanding their roles in immune surveillance. Furthermore, our system effectively demonstrates the opening and closing of gap junctions within an astrocyte network, induced by physiological environmental changes. The spontaneous neural activity observed through Ca^2+^ signals in neurons further validates the superior performance of TP‐FD‐FLIM volumetric projection imaging for in vivo neurodynamic research.

Although deep learning holds significant promises for denoising fluorescence images, it also presents several challenges, including dependence on training data, the risk of overfitting, and issues of interpretability. To address these challenges, we used HeLa cells labeled with mitochondrial probes to obtain a substantial number of high‐quality intensity, *g*, and *s* images for pre‐training the networks, which accelerates convergence and enhances generalization. The network was then further fine‐tuned on a smaller, task‐specific dataset, including brain vasculature imaging of zebrafish and mice, intracellular Ca^2+^ imaging, and microglia imaging in the zebrafish brain. To further mitigate overfitting, we employed various image augmentation techniques, such as flipping, rotation, intensity scaling. We applied CoBi loss for robust feature learning on the variable *g* dataset and used perceptual loss to enhance the visual accuracy of the *s* and intensity datasets. By utilizing the Adam optimizer and conducting over 500 iterations with a cosine scheduler, we minimized the MAE, achieving high fidelity in the resulting images.

In the future, a high‐performance rapid scanning device,^[^ ^16^ ^]^ the capability of simultaneously acquiring more phase images,^[^ ^22^ ^]^ combined with a volumetric excitation scheme utilizing light‐field modulation technology,^[^ ^51^, ^52^ ^]^ could significantly enhance acquisition speed, lifetime measurement precision, axial resolution, and information segregation, thereby improving system performance. Bessel‐based two‐photon fluorescence lifetime microscopy compresses and projects volumetric information onto a 2D plane, resulting in the loss of spatial axial position information. This limitation leads to reduced axial resolution and restricts the visualization of dense structures in vivo. An effective method to address this issue involves using phase‐shifted optical beatings of Bessel beams technology instead of axicon‐generated Bessel beams.^[^ ^53^, ^54^, ^55^ ^]^This approach can be integrated with volumetric two‐photon fluorescence lifetime microscopy to compensate for the loss in axial resolution. However, this method requires varying the beating frequency through a spatial light modulator to encode depth information, capturing over 100 frames to retrieve the depth information and improve axial resolution. Employing a deep learning framework can significantly reduce the number of frames needed while still achieving 3D volumetric imaging with high spatial resolution (both lateral and axial). The point spread function (PSF) engineering is an optical method designed to improve the optical sectioning capability.^[^ ^56^ ^]^ An extended depth of field (EDOF)^[^ ^57^ ^]^ and Tetrapod PSF^[^ ^58^ ^]^ in high‐throughput microscopy extend the acquisition depth while encoding spatial information, allowing for the recovery of 3D data from single snapshots. Additionally, integrating biomarker technology could expand the applications of TP‐FD‐FLIM volumetric projection imaging in biological research, enabling detailed exploration of neurodynamic processes at the molecular level. To address the challenges of biological tissue scattering, photon noise, and motion artifacts in vivo, adaptive optical technique^[^ ^59^ ^]^ and deep learning framework^[^ ^60^ ^]^ have been developed to improve the penetration depth by correcting wavefront distortion, suppressing noise, and recovering clear structures through enhanced spatiotemporal relations in optical microscopy data. Overall, our findings demonstrate that the combination of multi‐spatial dimensions (volumetric excitation), information dimensions (intensity, lifetime, and phasor components), and high‐speed information acquisition in two‐photon fluorescence lifetime volumetric projection microscopy allows for dynamic visualization and precise quantification of rapid biological processes. This makes it a crucial tool for investigating physiological phenomena, such as information exchange between neurons and glial cells in the central nervous system, as well as alterations in the brain microenvironment.

## 4 Experimental Section

### Animal Procedures and Sample Preparation

The animal experiments strictly adhered to the protocols and guidelines established by the Ethics Committee of Experimental Animals, Medical Department, Shenzhen University, China (Approval number: C202110‐01).

### Cell Culture

HeLa cervical cancer cells (Abiowell) and HL‐1 mouse cardiomyocytes (Abiowell) were cultured in Dulbecco's Modified Eagle's Medium (DMEM, Thermo Fisher Scientific) supplemented with 15% Fetal Bovine Serum (FBS, Cellorlab) and 1% penicillin–streptomycin (pen/strep, Thermo Fisher Scientific). The cells were plated in Corning cell culture flasks and cultured in an incubator at 37 °C with 5% CO_2_. The cells were passaged every other day to maintain optimal growth conditions.

### Probe preparation of MitoTracker Deep Red FM (Invitrogen) and Oregon Green 488 BAPTA‐1 (Invitrogen) Stock Solutions

80 µL of sterile DMSO was used to prepare a 1 mm stock solution of MitoTracker Deep Red FM. Similarly, 40 µL of sterile DMSO was used to prepare a 1 mm stock solution of Oregon Green 488 BAPTA‐1. The prepared stock solutions were aliquoted into multiple tubes, each containing 5 µL, to be used as reserve solutions.

### HeLa Cell Mitochondrial Staining

HeLa cells were plated on glass‐bottom culture dishes (Biosharp) and stained after complete adherence. MitoTracker Deep Red FM was used to stain the mitochondria. Prior to stain, the cells culture medium was removed, and the cells were washed with Phosphate buffer solution (PBS) 1–2 times. Then, 1 µL of 1 mm MitoTracker Deep Red FM was diluted with 1 mL PBS to a final concentration of 1 µm. This 1 µM PBS solution containing the probe was added to the culture dish, and the cells were incubated at 37 °C for 20 min. After incubation, the probe‐containing solution was removed, and the dish was washed with PBS 2–3 times, followed by the addition of 1 mL PBS for subsequent imaging.

### HL‐1 Cardiacmyocyte Calcium Measurement

After serum starvation for 4 h, HL‐1 cardiomyocytes were loaded with 5 µM Oregon Green 488 BAPTA‐1 (Invitrogen), which was thoroughly mixed with 20% poloxamer 407 before use. The cells were incubated at 37 °C with 5% CO2 for ≈40 min to complete the staining. The cells were then gently washed with PBS 2–3 times, followed by the addition of 1 mL HEPES‐buffered physiological saline for imaging. During the imaging process, 10 nm histamine was added to induce intracellular calcium ion oscillations.

### Zebrafish Imaging

The transgenic zebrafish lines used in this study were Tg (kdrl: eGFP) and Tg (pu1: eGFP), sourced from the China Zebrafish Resource Center. The zebrafish were raised in an E3 solution containing 0.003% N‐phenylthiourea (Sigma) to inhibit pigmentation starting at 20 h post‐fertilization. Prior to live imaging, the zebrafish were anesthetized with 600 µm Tricaine (Sigma, Cat. #E10521) and securely mounted in 1% low‐melting‐point agarose (NuSieve GTG, Cambrex BioScience, Cat. #50080) to minimize movement‐induced artifacts during the imaging process.

### Mice Brain Imaging

Prior to the experiments, animals were prepared according to established standard procedures. Male C57 mice (8 to 10 weeks old, weighing 20 to 21 g) were obtained from the Guangdong Medical Laboratory Animal Centre and used for in vivo high‐speed volumetric two‐photon fluorescence lifetime imaging experiments. Detailed cranial window procedures have been previously documented. Briefly, the mice were anesthetized using a gaseous anesthesia system (Matrix VIP 3000, Midmark) with 1.5% to 2% isoflurane. A heating blanket was employed to maintain a constant body temperature of 37 °C throughout the procedure. After removing the fur and scalp, a small section of the skull (≈2 mm in diameter) was carefully excised using a precision dental drill. A glass cover slip and a custom‐made titanium alloy ring were then securely affixed to the cranial window using dental cement. For cerebral vascular imaging, a 200 µL aqueous solution of FITC fluorescent probes was injected via the orbit to label the brain vasculature. For astrocyte imaging, 100 µL of SR101, diluted to 3.3 mg/mL, was administered via orbital injection. Imaging was conducted after a 3 h waiting period. For calcium measurement in neurons within the mouse cortex, the heating pad and the mouse were transferred to a stereotaxic instrument, and the syringe was adjusted to the appropriate position along the Z‐axis. The needle was positioned at the bregma injection area, and 5 nL of OGB‐1 calcium indicator (200 µm) was injected into specific locations within the visual cortex at a rate of 1 nL min^−1^ (X: −2.80 M/L, Y: −3.02 A/P, Z: −0.1 D/V; X: −2.80 M/L, Y: −3.02 A/P, Z: −0.2 D/V; X: −2.80 M/L, Y: −3.02 A/P, Z: −0.3 D/V; X: −2.65 M/L, Y: −2.88 A/P, Z: −0.09 D/V; X: −2.65 M/L, Y: −2.88 A/P, Z: −0.16 D/V; X: −2.65 M/L, Y: −2.88 A/P, Z: −0.25 D/V). After the injection, the cover slip was used to encapsulate the site, and the mouse head fixture was securely fixed in place for 1 h before imaging. At the end of the experiment, the mice were humanely euthanized under deep anesthesia through cervical dislocation.

### Optical Setups and Analog Signal Processing

For the two‐photon fluorescence lifetime volumetric projection microscopy (Figure S1, Supporting Information), a broadly tunable femtosecond laser (pulse width: 100 fs, repetition rate: 80 MHz, Chameleon Discovery, Coherent, 680–1300 nm) provided excitation pulses for two‐photon fluorescence imaging. The intensity was controlled by a laser controller, a half‐wave plate, and a PBS. After expansion and collimation in the optical 4f system, the laser pulse was directed to a beam‐switching device, which used flip mirrors to alternate between the Gaussian and Bessel modules. In the Bessel module, an axicon (AX251‐B, Thorlabs) generated Bessel beams with extended DOF, which were then transformed into halos with varying radii. Subsequently, the optical ring was relayed through an optical 4f system to a custom‐built two‐photon laser scanning microscope. The conventional two‐photon microscopy setup comprised a pair of galvo scanners (GVS002, Thorlabs), a scan lens (SL50‐2P2, Thorlabs), a tube lens (TTL200MP, Thorlabs), and an objective lens (UPLSAPO 60×, 1.2 NA, Olympus; Plan Apo Lambda 20×, 0.75 NA, Nikon; or XLPLN25XWMP2 25×, 1.05 NA, Olympus). The two‐photon excitation fluorescence (2PEF) was collected by the objective lens, reflected by a long‐pass dichroic mirror (ZT670rdc‐xxrxt, Chroma), filtered through short‐pass filters (ET680sp‐2p8, Chroma) and bandpass filters to eliminate residual excitation, and detected by a photomultiplier tube (PMT2101/M, Thorlabs) through a collection lens.

The analog signal processing module within the volumetric two‐photon fluorescence lifetime microscope efficiently utilized the fluorescence signal from the PMT, along with the 80 MHz reference signal from the femtosecond pulse laser, to concurrently generate intensity, lifetime, and phasor data. The 2PEF signal was detected by a PMT with a transimpedance amplifier. The output voltage signal was filtered using a LPF(BLP‐90+, Mini‐Circuits) and separated into DC and RF components using a bias tee (ZFBT‐282‐1.5A+, Mini‐Circuits). The DC signal was captured by a DAQ card (PXIe‐6115, National Instruments), enabling reconstruction of the intensity image. Additionally, the RF signal was amplified using a LNA (ZX60‐P103LN+, Mini‐Circuits) and divided into four separate paths using a four‐way power splitter (ZSC‐4‐3+, Mini‐Circuits). Concurrently, the 80 MHz reference signal from the laser was filtered through an LPF to eliminate higher harmonics, then amplified by an LNA and divided into four signal paths using another power splitter. A pair of phase shifters (JSPHS‐150 equipped with TB‐152+, Mini‐Circuits) introduced a 2π phase shift to each reference signal path. These phase shifts were independently adjusted through bias voltages generated by a DAQ card (PXIe‐6363, National Instruments). The four phase‐shifted reference signals are amplified by LNAs and directed toward four RF mixers (ZAD‐3H+, Mini‐Circuits). Each mixer received a reference signal path at its local oscillator (LO) port and a PMT signal path at its RF port, generating a mixed signal at its intermediate‐frequency (IF) port. The IF signals from each mixer were filtered to DC via an LPF (EF506, Thorlabs) and measured by a separate DAQ card (PXIe‐6363, National Instruments). Ultimately, the intensity image and four corresponding mixer images were simultaneously acquired, ensuring synchronized data acquisition and minimizing the impact of PMT timing jitter. Fluorescence lifetime images and phasor plots were promptly generated through fundamental matrix operations.

### Calibrations

The TP‐FLIM module was benchmarked by repeatedly measuring the lifetimes of rhodamine B and rhodamine 6G (Figure S3, Supporting Information). Using the correct fluorescence lifetime values as standards, the study obtained accurate phase‐shift voltage values and error compensation parameters. The results indicated that the measured fluorescence lifetimes of rhodamine 6G and rhodamine B were ≈4000 and 1700 ps, respectively, which were close to their typical values. These calibrations were also applied to the phasor plots. A detailed description is provided in Note S2 (Supporting Information).

### Advanced U‐Net Model for FD‐FLIM Image Reconstruction

In this study, the study tailored a U‐Net‐based model for FD‐FLIM, enhancing image reconstruction from 2 to 10 µs exposures by integrating CBAM into each U‐Net layer.^[^ ^61^, ^62^ ^]^ This approach improved focus on the key features crucial for managing the dynamic range in FD‐FLIM.

The model architecture employs an encoder–decoder framework. The encoder's convolutional layers and max pooling capture detailed fluorescence decay dynamics at multiple scales, whereas the decoder up‐samples and uses skip connections to ensure precise image localization. To address the challenges of FD‐FLIM, the study utilized specific loss functions: CoBi loss for the variable *g* dataset to ensure robust feature learning, and perceptual loss for the *s* and Intensity datasets to enhance visual accuracy.^[^ ^63^, ^64^ ^]^ Optimization was performed using the Adam optimizer (learning rate = 0.0001, betas = (0.9, 0.999)) with a cosine scheduler across 500 epochs, aiming for the lowest MAE to achieve high fidelity. To improve robustness, image augmentation techniques were applied, including flipping and rotation transformations, to the datasets. Input and corresponding ground‐truth images were 256 and 512 pixels, respectively, with a training‐to‐testing ratio of 6:1. The model was implemented using PyTorch 1.9.0 and Python 3.8.3, running on an NVIDIA RTX 3080 GPU and an AMD Ryzen 5 5600X CPU under Windows 10.

### Data Analysis

All data analyses rely exclusively on raw images, whereas for the purpose of enhancing the visibility of morphological features during exhibitions, the images undergo meticulous adjustments in ImageJ, specifically targeting the dynamic range (brightness/contrast). The resulting lifetime images and phasor plots were precisely calculated utilizing g, s, and intensity images, and a comprehensive description of this process is provided in Note S1 (Supporting Information).

In Figures 2 and 5, all intensity and lifetime data were derived from the raw intensity and lifetime images using ImageJ. The intensity curves were pre‐processed through normalization, and the fluorescence lifetime distribution histograms were nonlinearly fitted with the Lorentzian (Cauchy) function, the fluorescence lifetime distribution results were presented as peak values along with the MSE (peak values / MSE).

In Figure 3D, the fluorescence intensity and lifetime time series data were extracted from five different cells in the image stacks using ImageJ. The *ΔF/F* represents the change in fluorescence intensity relative to baseline fluorescence (initial fluorescence intensity value), where *ΔF* represents the change of fluorescence intensity, and *F* is the baseline fluorescence intensity. The *ΔF/F* curve is nonlinearly fitted using a fifth‐order polynomial function.

In Figure 4C, the fluorescence intensity and lifetime time series were first extracted data from three ROIs in the image stacks (Movie S3) using ImageJ. The study then applied mean filtering (averaging with 4 neighbors on each side) and a second‐order smoothing polynomial fitting to smooth and normalize the intensity curve. In Figure 4D, Sample size (50) for each statistical analysis, and the unpaired Mann–Whitney test was used to assess significant differences.

Additionally, all statistical analyses pertaining to mean, standard deviation, and significant differences, along with visualization, are meticulously conducted using GraphPad Prism.

## Conflict of Interest

The authors declare no conflict of interest.

## Author Contributions

Y.L. and L.L. supervised the research and designed the detailed implementations. Y.L. built the system, performed the experiments, analyzed the data, and developed the control networks. X.X. constructed and tested the deep network, developed the Python and MATLAB analysis codes, performed simulations, and analyzed the imaging data. C.Z., X.S., S.Z., and X.L. provided the animal models, performed craniotomies on mice, and prepared the samples. J.G., R.H., J.Q., and L.L. supervised the data analysis and edited the manuscript. All authors contributed to writing the manuscript.

## Supporting information



Supporting Information

Supporting Information

Supporting Information

Supporting Information

Supporting Information

Supporting Information

## Data Availability

The data that support the findings of this study are available from the corresponding author upon reasonable request.
