# Supplementary material for: In Vivo Neurodynamics Mapping via High‐Speed Two‐Photon Fluorescence Lifetime Volumetric Projection Microscopy
Source: Adv Sci (Weinh). 2024 Dec 23;12(7):2410605. doi: 10.1002/advs.202410605 (PMC11831470; doi:10.1002/advs.202410605)
Supplement: Supplementary file 1 — Supporting Information [file ADVS-12-2410605-s003.docx]

Supporting Information

*In Vivo* Neurodynamics Mapping via High-Speed Two-Photon Fluorescence Lifetime Volumetric Projection Microscopy

Yanping Li, Xiangcong Xu, Chao Zhang, Xuefeng Sun, Sisi Zhou, Xuan Li, Jiaqing Guo, Rui Hu, Junle Qu, and Liwei Liu*

**This PDF file includes:**

Supplementary Text

Figures S1 to S9

Movies S1 to S5

References (1 to 6)

Supplementary Note 1 Principle of frequency domain two-photon fluorescence lifetime microscopy (TP-FD-FLIM)

In two-photon frequency domain fluorescence lifetime microscopy (FD-FLIM), the fluorescence signal is detected by a photomultiplier tube (PMT) equipped with a transimpedance amplifier (PMT1001/M, Thorlabs). The instrument response function’s (IRF) of this setup has a full width at half maximum (FWHM) of 300 ps, which is significantly shorter than the lifetime values of most fluorophores we considered in this study, allowing us to ignore the IRF in the mathematical derivations for simplification. Specifically, after separating the direct current (DC) and radio frequency (RF) components of the PMT signal using a bias tee, the fluorescence intensity image can be generated from the DC component of the signal. This DC component represents the average fluorescence intensity detected over time and can be mathematically expressed as follows:

$V_{DC}=\frac{BP^{2}c}{T}+O_{B}$ (1)

Where *P* is the excitation power, *c* is the fluorophore concentration, *T* is the modulation period of the Femtosecond pulse laser, and *B* and *O_B_* are the the conversion loss and offset from the bias tee’s respectively. And the output of the mixers’ IF ports (the mixing results of RF signal and local oscillator) can be written as:

(2)

Where is the phase shift, *a_i_* is the intensity-weighted fractional contribution of the fluorophore with lifetime *τ_i_* ($\sum_{i} a_{i}=1$), *ω* is the angular modulation frequency, and *M* and *O_M_* are the conversion loss and offset from the mixer’s RF to IF ports, respectively. The average lifetime is calculated as

$\tau\text{=}\frac{1}{\omega}\frac{V_{IF}(0)-V_{IF}(\pi)}{V_{IF}(0.5\pi)-V_{IF}(1.5\pi)}$ (3)

And the components of the phasors are obtained as

$$g=\frac{B}{2M}\frac{V_{IF}(0.5\pi)-V_{IF}(1.5\pi)}{V_{DC}-O_{B}}=\sum_{i} a_{i}\frac{1}{1+(\tau_{i}\omega)^{2}}$$

$s=\frac{B}{2M}\frac{V_{IF}(0)-V_{IF}(\pi)}{V_{DC}-O_{B}}=\sum_{i} a_{i}\frac{\tau_{i}\omega}{1+(\tau_{i}\omega)^{2}}$ (4)

So, the average lifetime is also calculated as

$\tau\text{=}\frac{s}{g\omega}\approx\frac{\sum_{i} a_{i}\tau_{i}}{\sum_{i} a_{i}}=\sum_{i} a_{i}\tau_{i}$ (5)

In the phasor transformation, when the lifetime is a mono-exponential, the lifetime can be written as:

$\tau\text{=}\sqrt{\frac{1}{g}-1}/\omega$ (6)

The detailed principle introduction of FD-FLIM can be seen https:/doi.org/10.6084/m9.figshare. 14599863

Supplementary Note 2 Calibration of TP-FD-FLIM

We adopt the Rhodamine B with a known lifetime of 1.7 ns for system calibration. To ensure the accuracy and precision of lifetime measurements across various excitation wavelengths and to compensate for the wavelength dependency of PMT sensitivity, it is imperative to repeat the calibration process for each individual excitation wavelength utilized. Firstly, considering a fluorescence sample with a known lifetime$\tau$, we calibrated all four mixing signal paths according to the relationships between the phase shift and the bias voltage of the phase shifter. In the FD-FLIM, the mixers' outputs are low-pass filtered to eliminate all the harmonics, therefore, the filtered signals are pure DC voltages:

(7)

The curve about the relations between *V_b_* and *V_IF_* can be obtained, and the maximal and minimal values:

$\begin{matrix} V_{IF,\max}=\frac{P^{2}Mc}{T}\frac{1}{\sqrt{1+(\tau\omega)^{2}}}+O_{M} \\ V_{IF,\min}=\frac{P^{2}Mc}{T}\frac{-1}{\sqrt{1+(\tau\omega)^{2}}}+O_{M} \end{matrix}$ (8)

And then,

$\begin{matrix} V_{IF,\max}\text{+}V_{IF,\min}\text{=2}O_{M} \\ V_{IF,\max}\text{-}V_{IF,\min}\text{=}\frac{2P^{2}Mc}{T}\frac{1}{\sqrt{1+(\tau\omega)^{2}}} \end{matrix}$ (9)

The phase calibration curve can be obtained,

(10)

By following the above steps, phase shift of 0, 0.5π, π, 2π can be introduced by applying corresponding bias voltage *V_b_* according to the calibration curve , as shown in **Figure S2(b)and S2(c)**.

Second, the coefficient *M/B* and the offset *O_B_* in Eq. (4) should be determined in the calibration process. We obtained the offset *O_B_* by turning off the laser beam while measuring *V_DC_*. According to Equation (1) and Equation (9), we get

$\frac{M}{B}=\frac{V_{IF,\max}-V_{IF,\min}}{2(V_{DC}-O_{B})}\sqrt{1+(\tau\omega)^{2}}$ (11)

Which are used in the lifetime and phasor calculation.

Furthermore, we need to calibrate the DC offset difference across the four mixers. During this process, we turn off the laser, and measure the *V_IFi_* from each of four mixers:

$\begin{matrix} V_{IF1}=O_{M1}=O(P_{RF})+d_{1} \\ V_{IF2}=O_{M2}=O(P_{RF})+d_{2} \\ V_{IF3}=O_{M3}=O(P_{RF})+d_{3} \\ V_{IF4}=O_{M4}=O(P_{RF})+d_{4} \end{matrix}$ (12)

The *O(P_RF_)* represents a function of the RF signal’s power *P_RF_*, and is consistent across the four mixers. And we can get two calibration parameters, *d_13_* and *d_24_*, as:

$\begin{matrix} d_{13}=d_{1}-d_{3}=V_{IF1}-V_{IF3} \\ d_{24}=d_{2}-d_{4}=V_{IF2}-V_{IF4} \end{matrix}$ (13)

After calibration, the DC offset differences among the mixers can be compensated using *d_13_* and *d_24_* in the FD-FLIM system, and the lifetime can be calculated as:

$\tau\text{=}\frac{1}{\omega}\frac{V_{IF}(0)-V_{IF}(\pi)-d_{13}}{V_{IF}(0.5\pi)-V_{IF}(1.5\pi)-d_{24}}$ (14)

And the phasor components as:

$$g=\frac{V_{IF}(0.5\pi)-V_{IF}(1.5\pi)-d_{24}}{2(V_{DC}-O_{B})(M/B)}$$

$s=\frac{V_{IF}(0)-V_{IF}(\pi)-d_{13}}{2(V_{DC}-O_{B})(M/B)}$ (15)

All the calibration procedures described in this section can be performed though the LabVIEW program, which draws on the sub-VI of program <https://github.com/yzhang34/Instant-FLIM-Control.git> and <https://github.com/yzhang34/Instant-FLIM-Analysis.git>.

Supplementary Note 3 Axicon-based Bessel module in TP-FD-FLIM volumetric projection imaging

In traditional two-photon laser-scanning microscopy, a Gaussian beam is used to excite the sample at the focal point. However, due to the diffraction properties of the Gaussian beam, it rapidly widens above and below the focal plane, necessitating the acquisition of a stack of images at different depths. This requirement reduces the acquisition speed for volumetric samples. In this research, we adopt an axicon to generate a nondiffractive beam (Bessel beam) from Gaussian beams, which extends the depth of field (DOF) while maintaining lateral resolution. For the axicons (shaped as a cone), the angel between sloped and plane facesis defined as $\alpha$. And when a plane wave is incident upon an axicon, it is deviated towards the optical axis by an angle$\beta$, and defined as $\beta\text{=sin}^{-1}(n\sin\alpha)-\alpha$, where *n* is the refractive index of the axicon. All rays are deviated towards the optical axis z with the same azimuthal angle, therefore, the intensity is modulated by an interference pattern, resulting in a zero-order Bessel function that features an intense central lobe. The intensity distribution of the Gaussian beam transformed by the axicon can be expressed according to the Fresnel diffraction integral, using the stationary phase approximation^[1]^:

$I(r,z)=I_{0}\frac{4\pi^{2}\beta^{2}z}{\lambda}\exp(\frac{-2\beta^{2}z^{2}}{w^{2}_{0}})J^{2}_{0}(\frac{2\pi r\beta}{\lambda})$ (16)

Where *I_0_* is the intensity at the center of the incident beam, $\beta$ is the deviation angle of the axicon, $\lambda$ is the wavelength, *w_0_* is the initial Gaussian beam width, *z* is the optical axis, and *r* is the radial coordinate. We define the lateral resolution of Bessel beam as the size of the central lobe^[2]^:

$\rho\text{=}\frac{2.4048\lambda}{2\pi\beta}$ (17)

Considering the NA of objective, the lateral resolution of the generated Bessel focus is given as^[3]^

$\rho\text{=}\frac{2.4048\lambda f_{obj}}{2\pi\beta R_{pupil}}=\frac{2.4048\lambda}{2\pi\beta NA_{\text{eff}}}$ (18)

Here, $NA_{\text{eff}}=\frac{R_{pupil}}{f_{obj}}$is the effective numerical aperture of the Bessel focus scanned on the sample, which depends on the annular radius on the pupil plane of the objective$R_{pupil}=mf\sin\beta$, $f_{obj}$ is the effective focal length of the objective, *m* is the magnification from the frequency plane of Bessel beam to the pupil of the objective, *f* is the focal length of the lens after the axicon.

We can also extract the effective DOF, *L*, which is the on-axis distance along the central lobe that is intense enough to produce a contrasted fluorescence signal. We define *L* as the full width at half-maximum of signal point-spread function along the optical axis. Furthermore, the effective DOF is directly proportional to the squared intensity, *I (r=0, z)* in two-photon fluorescence^[2]^:

$L=C\left(\frac{w_{0}}{\beta}\right)$ (19)

After passing through the objective, the DOF can be expressed as:

$L=\left(\frac{f_{obj}}{mf}\right)\times\frac{C\times w_{0}}{\tan\beta}$ (20)

Where the constant *C* has the value of 0.58 for two-photon set-ups. From this relation, we can independently control DOF and the lateral resolution of the beam by varying the angle of the axicon and the width of the incident beam.

Supplementary Note 4 Performance evaluation of TP-FD-FLIM volumetric projection microscopy

To confirm the accuracy of fluorescence lifetime and phasor measurements in TP-FD-FLIM, we acquired lifetime images, phasor plots, and fluorescence lifetime distribution histogram for three fluorophores with single-exponential decays, as shown in **Figure S3**. In the phasor plots, the phasors of these single-exponential fluorophore all located on the universal semicircle, and their lifetime results are consistent with the standard values: Rhodamine B, 1.71±0.06 ns; Rhodamine 6G, 3.62±0.21 ns; Sodium Fluorescein, 4.02±0.27 ns. Additionally, the temporal resolution was determined by measuring the lifetime of the SHG signal from a urea crystal specimen, which approaches the IRF of the system (300 ps). This result demonstrates that our FD-FLIM module is capable of accurately measuring short lifetimes and differentiating small lifetime variations.

We estimated the lateral resolution and DOF of TP-FD-FLIM volumetric projection system using 200-nm-diameter fluorescent beads (f8888, Thermo Fisher), as shown in **Figure S5(a)**. During TPEF imaging with 840 nm excitation and a 20×, 0.75NA objective, the average lateral resolution, determined as the FWHM of the Gaussian function fitted to the experimental data, is approximately 550 nm for the Gaussian and Bessel foci. The DOF is 35 μm for the Bessel beam and 2.5 μm for the Gaussian beam, respectively. When using a 40×, 0.80NA objective, the lateral resolution is approximately 510 nm for both the Gaussian and Bessel foci, with the DOF being 12 μm for Bessel beam and 1.8 μm for Gaussian beam. The lateral resolutions were calculated by averaging the FWHM of fluorescent beads at various depths. We further analyzed the lateral resolution (using the 20×, 0.75NA) at different axial positions within the DOF range (**Figure S5(b)**). The results reveal that the resolution decreases at the end of the Bessel beam due to the uneven intensity distribution of the Bessel beam along the axial direction. We compared the lateral resolution of the Bessel and Gaussian beam across the entire DOF and found that the average FWHM of the fluorenscent beads was nearly identical for both beams.

To further validate the accuracy of lifetime and phasor measurement in TP-FD-FLIM volumetric projection imaging, we obtained the lifetime images, phasor plots, and fluorescence lifetime distribution histogram for two fluorophores with single-exponential decays, which were excited by Bessel beams, as shown in **Figure S5(c)**. The results demonstrate that the extended DOF of the Bessel beam does not affect the accuracy of the lifetime measurements. Specifically, the phasors of two fluorophore are located on the universal semicircle in the phasor plots, and fluorescence lifetime histograms feature the same distribution patterns as those observed in Gaussian-based TP-FD-FLIM: Rhodamine B, 1.73±0.08 ns; Sodium Fluorescein, 3.96±0.23 ns.

Supplementary Note 5 Quantification of performance for deep learning network

We used four metrics to evaluate the performance of our networks: peak signal-to-noise ratio (PSNR), the structural similarity index measure (SSIM), Root mean squared error (RMSE), and mean absolute error (MAE)^[4-6]^. Each metric was calculated as below:

$PSNR=10\times\log_{10}\left(\frac{R^{2}}{\sum_{i=1}^{W} \sum_{j=1}^{H} (U(i,j)-V(i,j))^{2}/(W\times H)}\right)$ (21)

$RMSE=\sqrt{\sum_{i=1}^{W} \sum_{j=1}^{H} (U(i,j)-V(i,j))^{2}/(W\times H)}$ (22)

$MAE=\sum_{i=1}^{W} \sum_{j=1}^{H} \left|U(i,j)-V(i,j)\right|/(W\times H)$ (23)

$SSIM=\left(\frac{2\bar{U}\bar{V}+C_{1}}{\bar{U}^{2}+\bar{V}^{2}+C_{1}}\right)\times\left(\frac{2\sigma_{UV}+C_{2}}{\sigma_{U}^{2}+\sigma_{V}^{2}+C_{2}}\right)$ (24)

Here, *W* and *H* represent the width and height of the ground truth image in the training step. *U* and *V* represent the ground truth image and the output of the network, respectively. $\bar{U}$, $\bar{V}$, $\sigma_{U}$, $\sigma_{V}$, and $\sigma_{UV}$are the averages, variances and covariance of *U* and *V*. The items *C_1_* and *C_2_* are small positive constants that stabilize each term ($C_{1}=(k_{1}L)^{2},C_{2}=(k_{2}L)^{2}$ , *L* is the dynamic range of the pixel-values, *k_1_* = 0.01 and *k_2_* = 0.03) by default. We then computed the performance of each metric for each architecture based on the output of the networks and the ground truth images, as shown in **Figure S6(b)**.

Supplementary Note 6 High-throughput volumetric projection imaging of vasculature in intact zebrafish and mouse brains

To demonstrate the performance of high-speed two-photon fluorescence lifetime volumetric projection microscopy, we acquired fluorescence intensity and lifetime volumetric projection images (400 μm × 400 μm × 35 μm volumes) of vasculature in transgenic zebrafish and mouse brain, excited by both Gaussian and Bessel foci (**Figure S7** and **Figure S8**). The 3D projection images based on the Gaussian beam were acquired by axial scanning with a step size of 1 μm for intensity image and 5 μm for lifetime image. The intensity images were color-coded to reveal depth information. During this process, it took 30 s to acquire a stack of 30 images, each with a resolution of 512 × 512 pixels and a pixel dwell time of 2 μs. This approach limits dynamic *in vivo* monitoring due to its relatively slow acquisition speed. In contrast, the two-dimensional projection of 3D volumetric imaging can be captured through single-frame scanning with Bessel beam excitation, which improve the imaging throughput by a factor of 30. In this study, we achieved a speed increase of over 30 times in TP-FD-FLIM volumetric projection imaging, which is attributed to the ratio of the DOF of the Bessel beam compared to the axial step size used with Gaussian beams. Additionally, we performed a comparative analysis of fluorescence lifetime measurements obtained from various axial positions using Gaussian beams. The results showed that the measurement error due to axial position variations was significantly smaller than the precision of the fluorescence lifetime measurements, confirming the consistency of the fluorescence lifetime information within the Bessel beam's focal depth range.


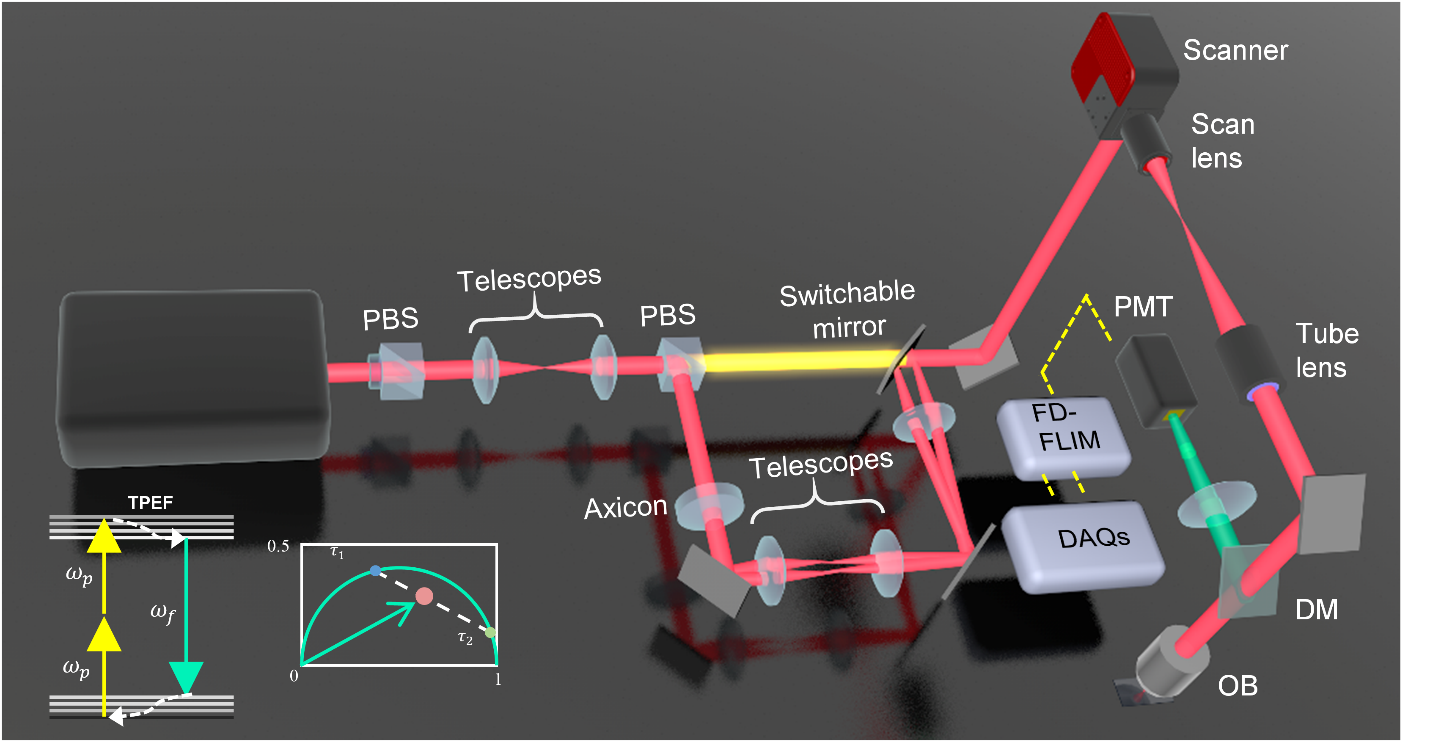


Figure S1. Schematic of the two-photon fluorescence lifetime volumetric projection microscopy. See complete details in Materials and Methods. Bottom left shows Jablonski diagrams of TPEF with its phasor transformation. DAQ, data acquisition system; DM, dichroic mirror; OB, objective; PBS, polarizing beam splitter; PMT, photomultiplier tube.


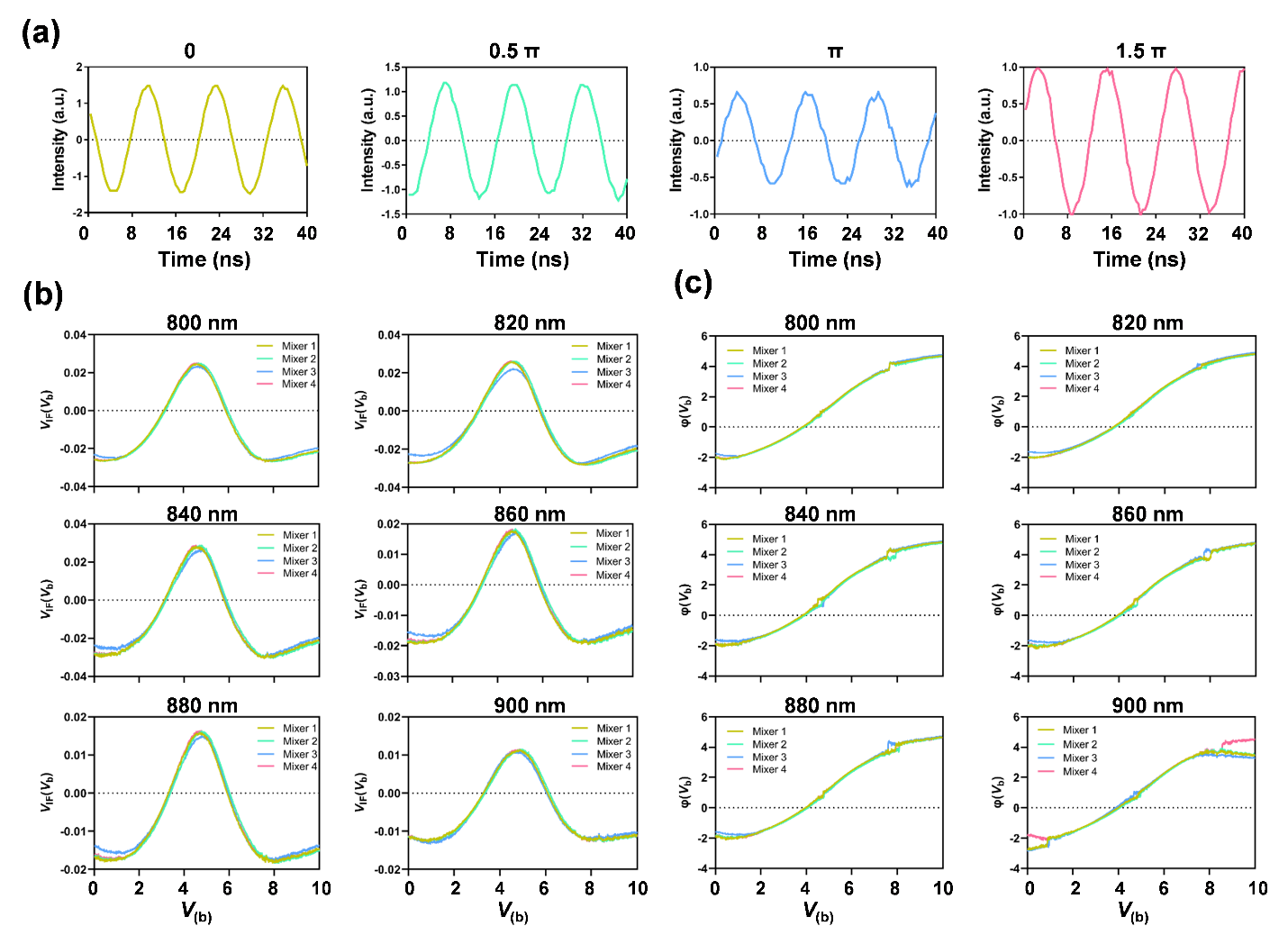


Figure S2. Analog signal processing and calibration in TP-FD-FLIM. (a) Four-channel phase-shift reference signals are generated by the Femtosecond pulse laser, the 80MHz signals are directed to the mixers’ LO ports after low pass filtering, amplifying, and splitting. (b) Calibration curves obtained by changing the bias voltage (Vb, from 0 to 10V) of four phase shifters and measuring the voltages (VIF) on the four mixers’ IF ports. (c) Phase calibration curves, generated from the raw data in (b) according to Equation (10). The experimental conditions: excitation wavelength at 800 nm, 820 nm, 840 nm, 860 nm, 880 nm, 900 nm; sample, Rhodamine B (1.7 ns); excitation power, 3 mW.


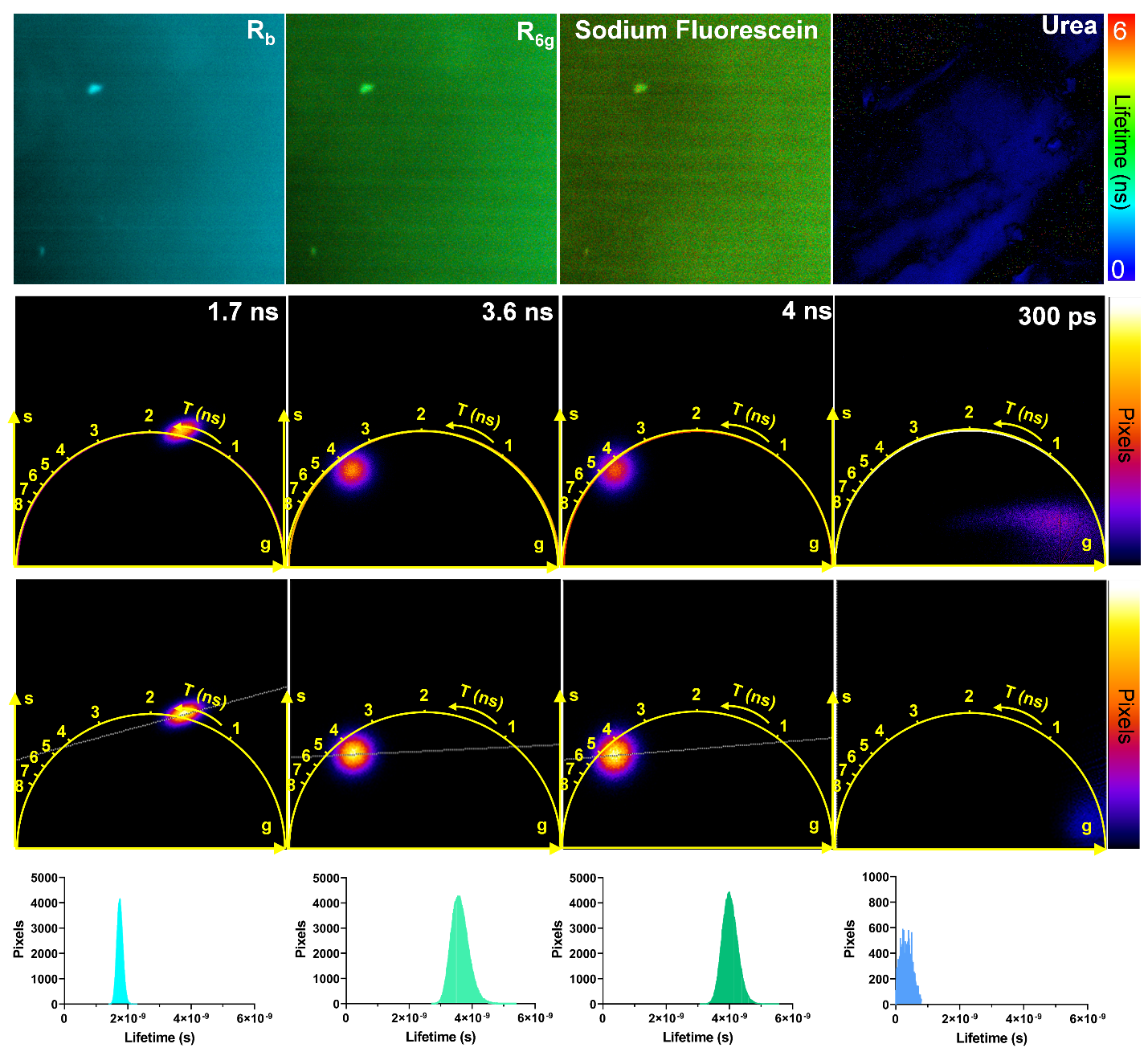


Figure S3. Fluorescence lifetime results of four samples. Fluorescence lifetime images (first line), phasor plot (second line) calculated by real-time signal processing, phasor plot drawn by ImageJ based on the phasor components *g* and *s* (third line), and a lifetime distribution histogram (fourth line) of three fluorescence lifetime standards in solution (including Rhodamine B, Rhodamine 6G, Sodium Fluorescein) and urea crystal (measuring the IRF).


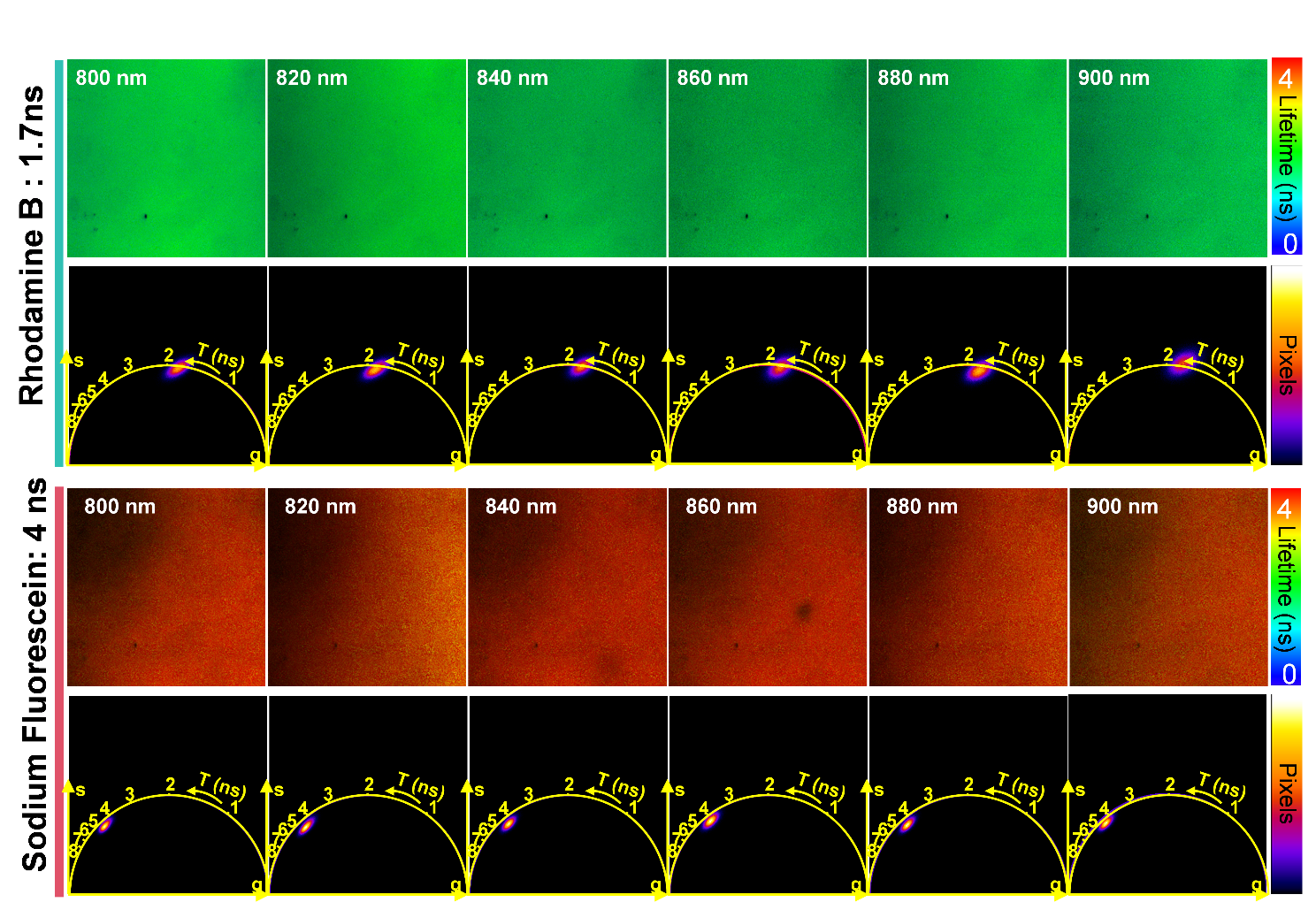


Figure S4. Fluorescence lifetime images and phasor plot of Rhodamine B and Sodium Fluorescein excited by different wavelengths (800 nm, 820 nm, 840 nm, 860 nm, 880 nm, 900 nm).


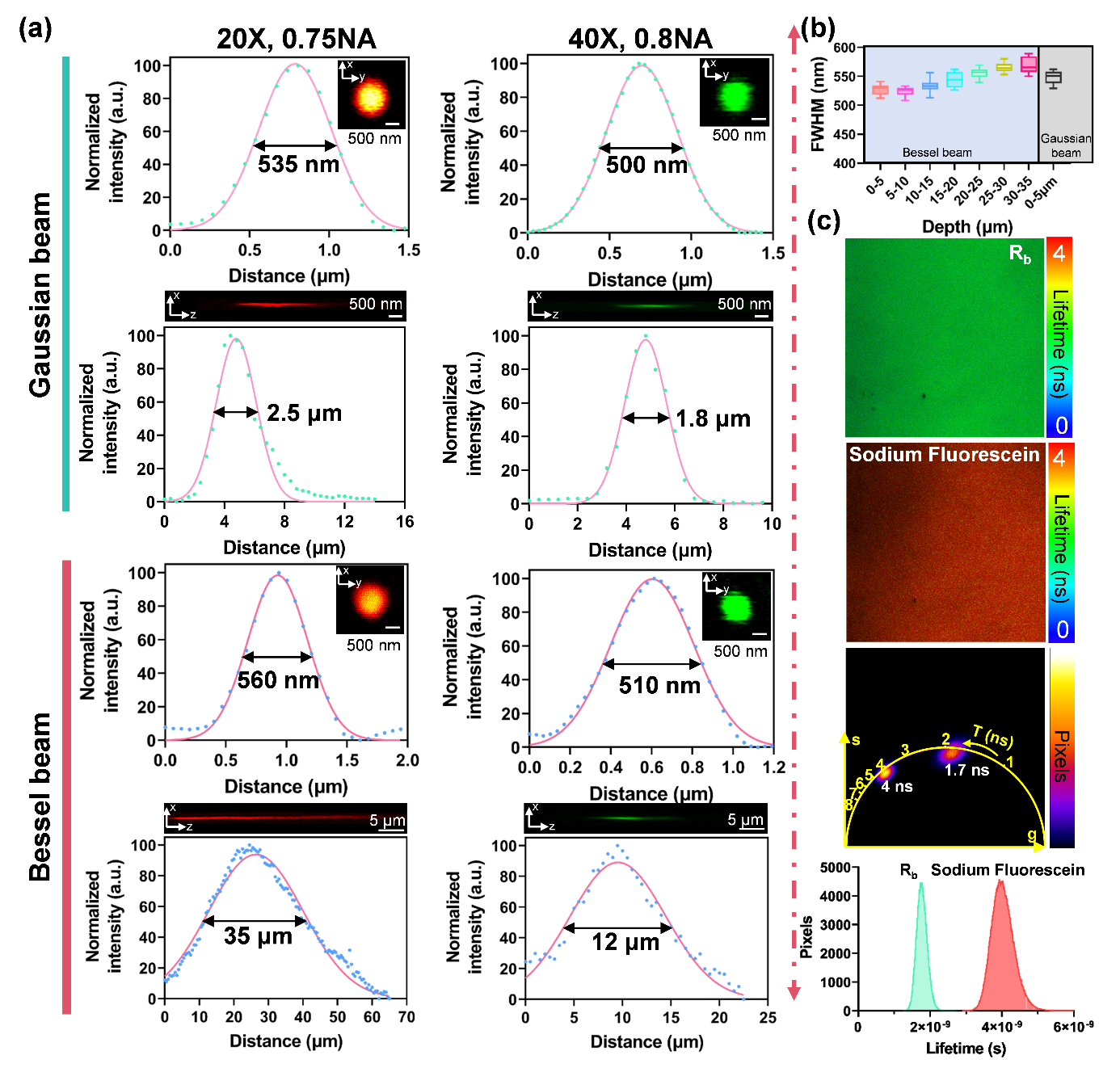


Figure S5. Performance evaluation of TP-FD-FLIM volumetric projection imaging. (a) Lateral resolution and DOF of TPEF imaging using the Gaussian and Bessel beams foci. Results from a single 0.2-μm-diameter bead. (b) Statistical results of the FWHM of fluorescent beads at different imaging depths in TPEF imaging excited by Bessel beam and Gaussian-based TPEF imaging. (c) Fluorescence lifetime images, phasor plot, and lifetime distribution histograms of Rhodamine B and Sodium Fluorescein excited by Bessel beams.


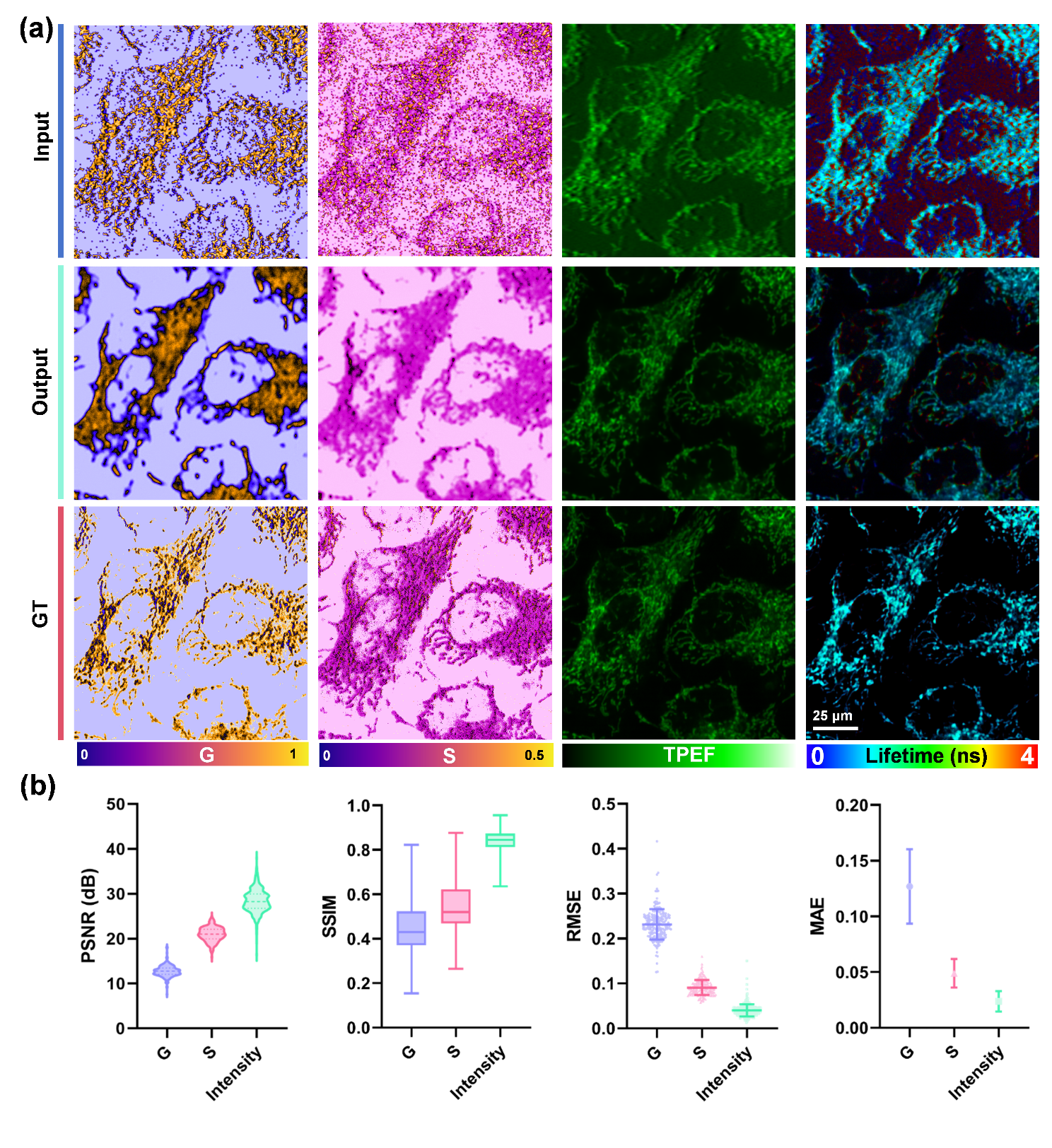


Figure S6. Analysis of the performance for deep learning network. (a) Fluorescence intensity and lifetime images of mitochondria marked by MitoTracker® Red in Hela cells, including input, output, and GT results. (b) Statistical analysis of image restoration quality assessment for *g*, *s*, and intensity images.


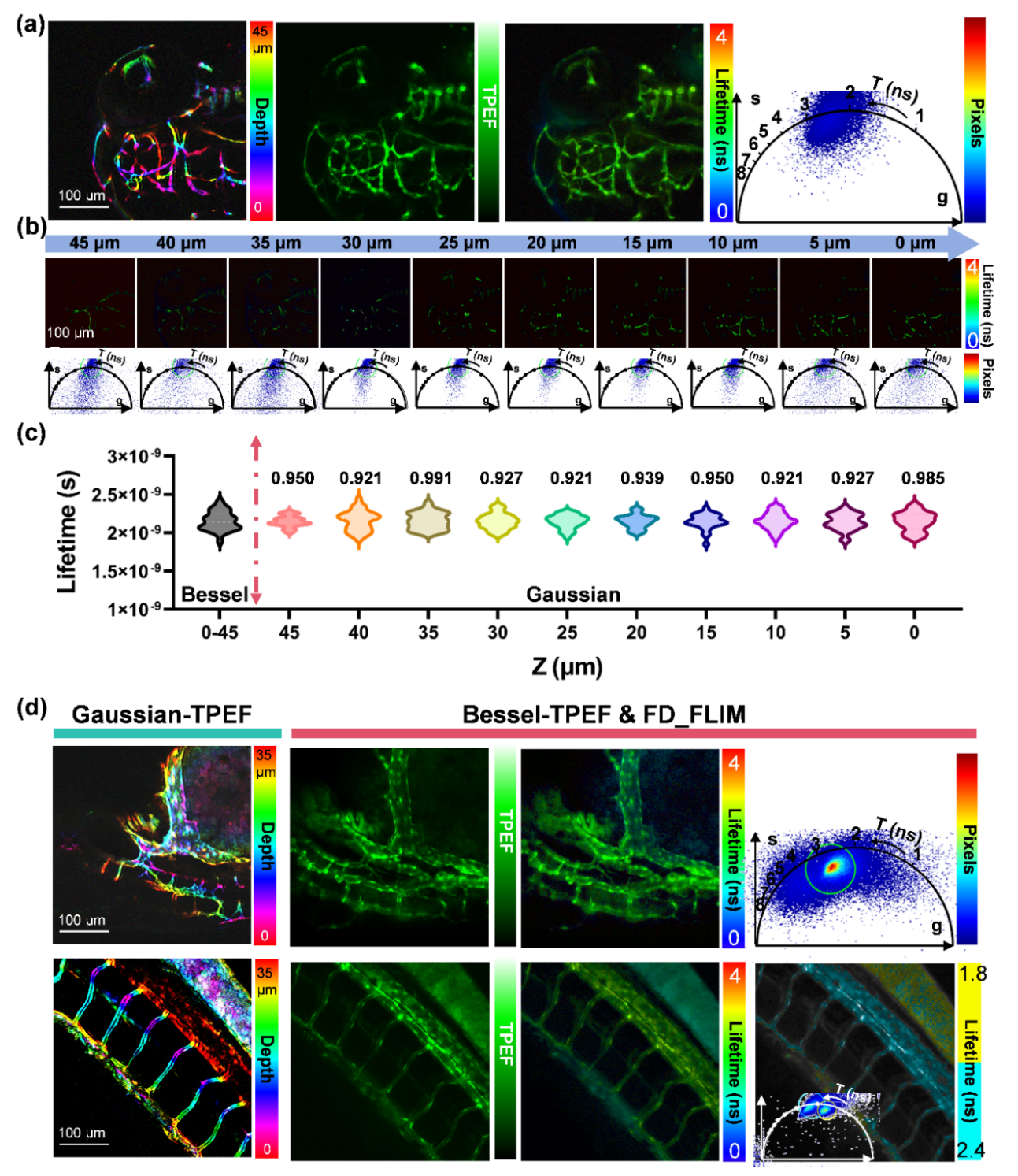


Figure S7. Two-photon intensity images and FLIM results of zebrafish vasculature expressing enhanced green fluorescence protein (eGFP). (a) The projection of a Gaussian two-photon intensity image stack, along with Bessel two-photon intensity, FLIM volumetric projection images, and phasor plot for zebrafish brain vasculature. (b) Gaussian two-photon FLIM images and phasor plots at different axial depths corresponding to (a). (c) Statistical results and significant difference analysis of Bessel and Gaussian two-photon fluorescence lifetime results obtained from (a) and (b). (d) The projection of Gaussian two-photon intensity image stack, as well as Bessel two-photon intensity, FLIM volumetric projection images, and phasor plot for zebrafish vasculature.


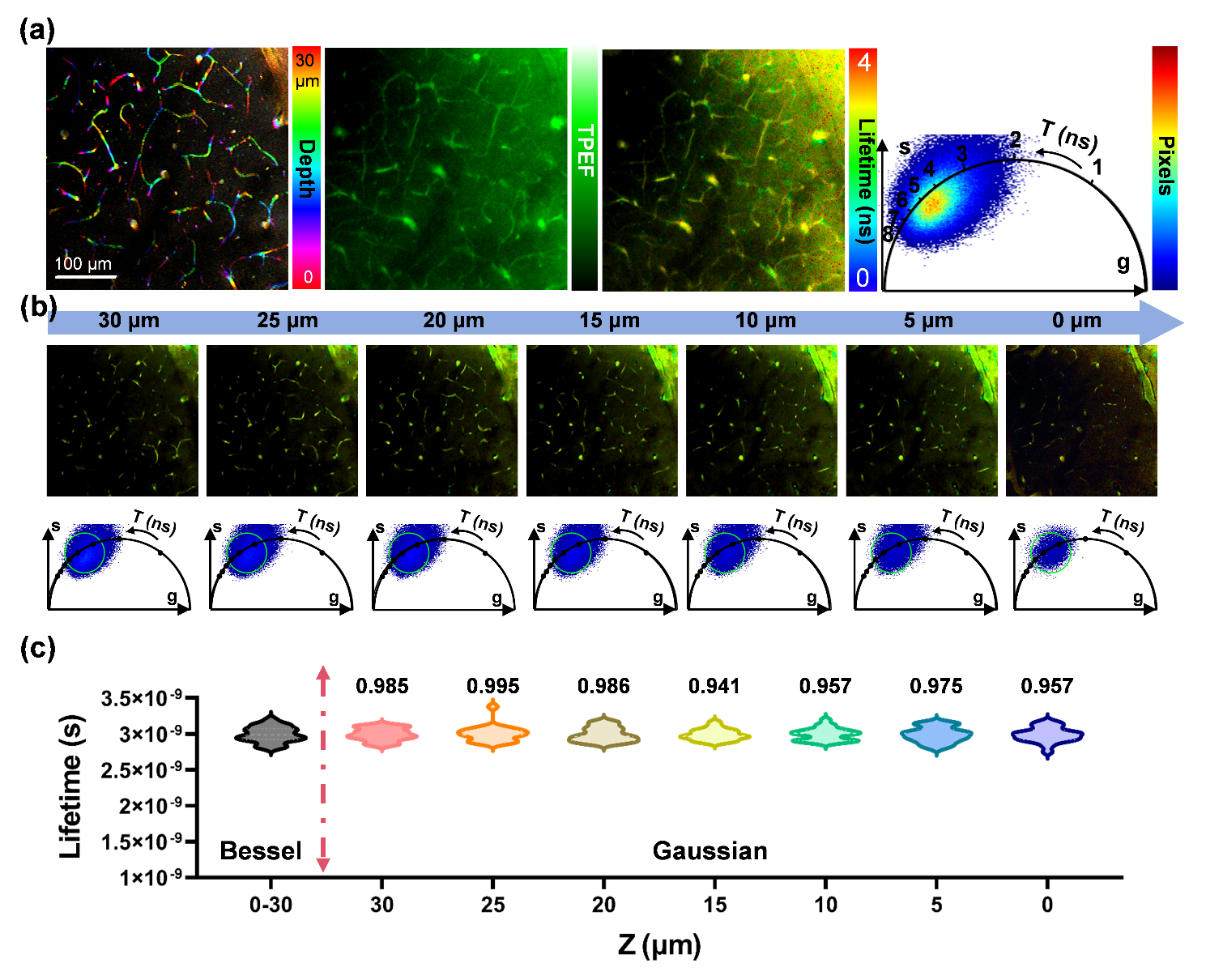


Figure S8. Two-photon intensity images and FLIM results of mouse brain blood vessels labeled with FITC. (a) The projection of a Gaussian two-photon intensity image stack, along with Bessel two-photon intensity, FLIM volumetric projection images, and phasor plot for mouse brain blood vessels. (b) Gaussian two-photon FLIM images and phasor plots at different axial depths for (a). (c) Statistical results and significant difference analysis of Bessel and Gaussian two-photon fluorescence lifetime results obtained from (a) and (b).


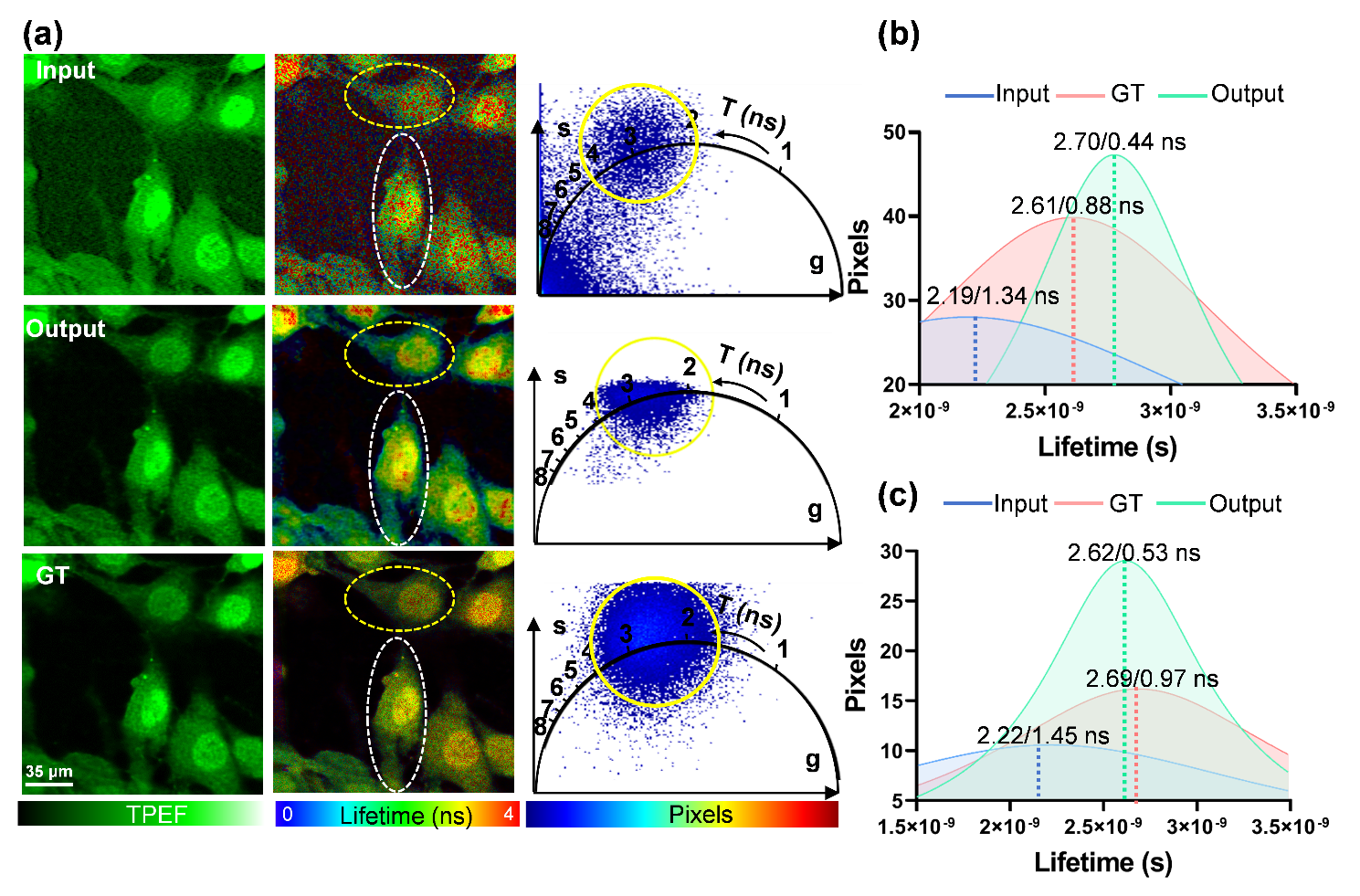


Figure S9. Fluorescence intensity images, lifetime images, phasor plots of intracellular Ca^2+^ concentration imaging. (a) A comparison between input and network output for fluorescence intensity, lifetime images, and phasor plots. (b) Histogram of the fluorescence lifetime distribution within yellow circle areas in (a). (c) Histogram of the fluorescence lifetime distribution within white circle areas in (a).

Movie S1. Dynamic monitoring of intracellular Ca^2+^ concentrations.

Raw fluorescence lifetime images and deep learning algorithm-powered fluorescence lifetime time-series results of intracellular Ca²⁺ concentrations over 90 seconds were measured using the high-speed FLIM volumetric projection system. Intracellular Ca²⁺, indicated by OGB-1, was excited with a femtosecond pulse laser at 820 nm using a 40× objective and was stimulated with histamine (at t=2 s). Arrowheads denote the moments when the intracellular calcium concentration rises.

Movie S2. Dynamic functional imaging of microglia in non-injury Zebrafish.

Fluorescence intensity and lifetime movies of microglia in non-injury zebrafish (*Tg (pu1: eGFP)*) brains were acquired over a duration of 250 seconds using the high-speed FLIM volumetric projection system. The microglia, indicated by arrowheads, exhibit small-scale movements while maintaining consistent fluorescence lifetime results.

Movie S3. Dynamic functional imaging of microglia in injury Zebrafish.

Fluorescence intensity and lifetime movies were obtained using the high-speed FLIM volumetric projection system and further enhanced with a deep learning algorithm. The region of interest (ROI) encompassing the microglia, denoted by arrowheads, was excited by an 800 nm femtosecond laser with high power (40 mW) for 5 minutes to induce thermal injury. The dynamic functional imaging results, including motion trajectories and fluorescence lifetime variations, reveal the biochemical response of zebrafish microglia to the injury.

Movie S4. Astrocytes functional imaging in mice brain.

Raw and deep learning algorithm-enhanced fluorescence intensity and lifetime time-series results of astrocytes in mouse brains (depth: 200-230 μm) were obtained using the high-speed FLIM volumetric projection system. Arrowheads denote the events related to the opening and closing of gap junctions.

Movie S5. Spontaneous calcium dynamics in mice brain

Raw and deep learning algorithm-enhanced fluorescence intensity and lifetime time-series results of Ca²⁺ in neurons within the mouse cortex (depth: 150-180 μm) were measured using the high-speed FLIM volumetric projection system. Arrowheads denote fluctuations in Ca²⁺ levels in single neurons.

Supplementary References

[1] W. Chen, X. Ge, Q. Zhang, R. G. Natan, J. L. Fan, M. Scanziani, N. Ji, Nature Methods **2024**.

[2] G. Thériault, Y. De Koninck, N. McCarthy, Opt. Express **2013**, 21, 10095.

[3] Y. Chen, C. Luo, S. Wang, Y. Li, B. Shen, R. Hu, J. Qu, L. Liu, Journal of Biomedical Optics **2024**, 29, 016501.

[4] Z. Lu, Y. Liu, M. Jin, X. Luo, H. Yue, Z. Wang, S. Zuo, Y. Zeng, J. Fan, Y. Pang, Nature Methods **2023**, 20, 735.

[5] C. Qiao, D. Li, Y. Guo, C. Liu, T. Jiang, Q. Dai, D. Li, Nature methods **2021**, 18, 194.

[6] N. Wijethilake, M. Anandakumar, C. Zheng, P. T. So, M. Yildirim, D. N. Wadduwage, Light: Science & Applications **2023**, 12, 228.
